# Morphological description and DNA barcoding research of nine *Syringa* species

**DOI:** 10.3389/fgene.2025.1544062

**Published:** 2025-02-26

**Authors:** Meiqi Zhang, Xiaoou Zhai, Lianqing He, Zhen Wang, Huiyan Cao, Panpan Wang, Weichao Ren, Wei Ma

**Affiliations:** ^1^ State Key Laboratory of Tree Genetics and Breeding, Northeast Forestry University, Harbin, China; ^2^ College of Landscape Architecture, Northeast Forestry University, Harbin, China; ^3^ Laboratory of Ornamental Plant Cultivation, Heilongjiang Forest Botanical Garden, Harbin, China; ^4^ College of Pharmacy, Heilongjiang University of Chinese Medicine, Harbin, China

**Keywords:** *Syringa*, DNA barcoding, ITS2, PSBA-TRNH, trnL-trnF, TRNL, species identification

## Abstract

**Introduction:**

*Syringa* plants are highly valued for their ornamental qualities. However, traditional morphological identification methods are inefficient for discriminating *Syringa* species. DNA barcoding has emerged as a powerful alternative for species identification, but research on *Syringa* DNA barcodes is still limited.

**Methods:**

This study employed a multi-locus strategy, combining the nuclear *ITS2* region with chloroplast genome regions *psbA-trnH*, *trnL-trnF*, and *trnL* to evaluate the effectiveness of *Syringa* DNA barcodes. The assessment involved genetic distance analysis, BLAST searches in NCBI, sequence character analysis, and phylogenetic tree construction, examining both individual and combined sequences.

**Results:**

The genetic distance analysis showed that the sequence combination of *ITS2* + *psbA-trnH* + *trnL-trnF* exhibited a variation pattern where most interspecific genetic distances were greater than intraspecific genetic distances. The Wilcoxon signed-rank test results indicated that, except for *psbA-trnH*, the interspecific differences of the *ITS2* + *psbA-trnH* + *trnL-trnF* sequence were greater than those of all single and combined sequences. BLAST analysis revealed that the identification rate for nine *Syringa* species using *ITS2* + *psbA-trnH* + *trnL-trnF* could reach 98.97%. The trait-based method also demonstrated that *ITS2* + *psbA-trnH* + *trnL-trnF* could effectively identify the nine *Syringa* species. Furthermore, the neighbor-joining (NJ) tree based on *ITS2* + *psbA-trnH* + *trnL-trnF* clustered each of the nine *Syringa* species into distinct clades.

**Discussion:**

The study ultimately selected the barcode *ITS2* + *psbA-trnH* + *trnL-trnF*, with an identification rate of 93.6%, as the optimal barcode for identifying nine species of *Syringa* trees. This combination proved to be highly effective in discriminating *Syringa* species, highlighting the potential of DNA barcoding as a reliable tool for species identification in *Syringa*. Future research could focus on expanding the sample size and exploring additional genetic markers to further enhance the accuracy and applicability of DNA barcoding in *Syringa* species identification.

## 1 Introduction

*Syringa*, a genus of deciduous shrubs or small trees, belongs to the Oleaceae family. With approximately 27 species globally, *Syringa* is predominantly found in East Asia, Central Asia, and Europe. They are renowned for their diverse flower colors and distinctive fragrance, which have made them a common choice for landscaping worldwide (Ming et al., 2007). Beyond their aesthetic appeal, *Syringa* species are also valued for the diverse chemical constituents found in their flowers, stems, leaves, roots, and fruits, which are used as premium raw materials in the medical and cosmetic industries (Wang et al., 2018). At the turn of the 21st century, the dried leaves of several *Syringa* species were officially recognized and incorporated into the standards of traditional Chinese medicinal materials. This recognition was based on the significant hypoglycemic, anti-inflammatory, antiviral, antioxidant, antitumor, and protective properties of *Syringa* leaves, particularly beneficial to the liver, heart, and nervous system (Zhang et al., 2006; Zheng and Guo, 2013; Zhao et al., 2016; Zhu et al., 2021). Since their inclusion in various pharmacopeias, research into the chemical composition and pharmacological effects of *Syringa* leaves has expanded, with comprehensive reviews detailing the chemical constituents and pharmacological activities of these species (Su et al., 2015), with active components such as phenylpropanoids and iridoids being employed in the treatment of gynecological inflammation, vomiting, diarrhea, cough, and bronchitis (Liu et al., 2020; Yang et al., 2021). The chemical composition varies significantly among different *Syringa* species, complicating the identification process during the procurement of raw materials for traditional Chinese patent medicines. This can lead to the adulteration of inferior or counterfeit products in the market, underscoring the need for efficient and accurate identification methods to support quality control and market regulation (Wang et al., 2003). Taxonomic studies on *Syringa* species have been complicated by long-term cultivation, outcrossing, and natural hybridization, resulting in unclear species boundaries within the genus. Correct and effective differentiation of *Syringa* species is therefore of paramount importance (Chen, 2006). Morphological identification, which requires specialized taxonomic knowledge and detailed descriptions of species morphology at various developmental stages, has several limitations. It may not fully capture potential genetic variations, especially in closely related species with intermediate and similar phenotypes, as well as recently diverged or hybrid-derived species (Chen et al., 2009; Hu et al., 2009; Lattier and Contreras, 2017). To address the challenges in the identification of *Syringa* species, in addition to traditional morphological identification, some studies have opted to use molecular methods for species determination. Research has identified nine new polymorphic microsatellite sequence markers for distinguishing common *Syringa* varieties (Juntheikki-Palovaara et al., 2013). Currently, there is a scarcity of research employing molecular methods for the identification of *Syringa* species. These limitations highlight the need for more reliable and effective methods for *Syringa* species identification, given their medicinal value and the varying conclusions drawn from earlier taxonomic studies.

DNA barcoding technology utilizes one or several standardized short DNA regions to identify taxonomic groups, providing a precise and rapid method for species identification (Fu et al., 2010; Cheng et al., 2011). This technology has been widely applied in the classification and evolutionary studies of various forest trees and medicinal plants, demonstrating high accuracy (Liu et al., 2019; Frigerio et al., 2021; Liu et al., 2021). Despite its widespread use, there is currently no consensus on the ideal barcode for *Syringa* species. One of the main challenges faced by barcodes is the identification of sister species. In forest trees, chloroplast genomic coding sequences such as *rbcL*, *matK* genes, intergenic spacers *psbA-trnH*, *trnL-trnF*, and introns *trnL* are commonly used for phylogenetic and kinship analyses (Bafeel et al., 2012; Liu et al., 2012; Yang et al., 2012; Yang et al., 2018; Cai et al., 2022; Wei et al., 2024). *MatK* and *rbcL* are two standard plant DNA barcode loci recommended by the Consortium for the Barcode of Life (CBOL). Numerous experiments have been conducted using these markers across various taxonomic units and species. However, the identification results have not been satisfactory. Some researchers have indicated that the universality and discriminatory power of *matK* primers are not ideal (Chase et al., 2007). In another research, it is emphasized that *matK* and *rbcL* are predominantly employed for taxonomic ranks above the genus level (Bi et al., 2020). In the analysis and identification of *Ligustrum lucidum* within the Oleaceae family, the DNA barcoding fragments *matK* and *rbcL* were utilized for species identification. The results indicated that both sequences exhibited low efficiency in species discrimination (Wang et al., 2023). To establish a DNA barcode suitable for the identification of *Syringa* plants, the *ITS* sequence from the nuclear genome was initially selected. However, it was found that the *ITS* sequence exhibited specific amplification bands during the amplification process and showed double peaks during sequencing, making it unsuitable for identification studies as a barcode for the genus *Syringa*. Additionally, the nuclear *ITS2* region is recognized as an effective barcode for species identification (Li et al., 2013; Zhu and Gao, 2014; Chen et al., 2016; Chen et al., 2019). Sequencing of the internal transcribed spacer *ITS2* region of ribosomal DNA has been used to determine the kinship of *Syringa* species in Northeast China (Li et al., 2010). The *ITS2* sequence, located between the 5.8S rRNA and 25S rRNA, was chosen. The *ITS2* sequence achieved a 100% amplification success rate in *Syringa* identification studies and demonstrated advantages in terms of variation, sequence quality, and high interspecific and intraspecific differentiation capabilities (Wang et al., 2015). Studies have shown that the *ITS2* sequence has a good distinguishing effect on plants in the genus *Hoya* (Xia et al., 2023). In the selection of chloroplast genomic sequences, in addition to *psbA-trnH* and *trnL-trnF*, the *trnL* intron, which has not been used in *Syringa* plant studies, was also included. The *trnL* intron is also a commonly used barcode sequence in molecular systematics, with a variation degree greater than that of mitochondrial gene sequences but significantly lower than the evolutionary rate of nuclear genes, often used for analyzing phylogenetic relationships at the interspecific and intraspecific levels within a genus. In the identification of five species of Phoebe, the *trnL* sequence was able to distinguish between *Machilus oreophila* and *Machilus pauhoi* (Qiao et al., 2019). Sometimes, the selection of plant barcodes cannot be limited to a single fragment; it is necessary to supplement with additional fragments according to the requirements. The use of a combination of multiple fragment markers is often required. In *Syringa* species, a single *psbA-trnH* sequence was found to be insufficient for distinguishing 33 *Syringa* samples, whereas the combined barcode of *psbA-trnH* and *trnC-petN* showed a higher identification rate for these samples. These findings highlight the need for further research on both single and combined DNA barcode sequences for *Syringa* species. This study employs four individual sequences *ITS2*, *psbA-trnH*, *trnL-trnF*, and *trnL* as well as 11 combined sequences for analysis. The aim is to compare different analytical methods and to explore whether combined sequences can enhance the identification capability of *Syringa* species. Ultimately, this study seeks to identify the optimal DNA barcode combination for the accurate identification of *Syringa* species.

## 2 Results and analysis

### 2.1 Morphological characteristic analysis

The morphological characteristics of three leaf traits (leaf shape, leaf base shape, leaf color) and four flower traits (flowering period, inflorescence shape, petal type, flower color) were statistically analyzed for nine *Syringa* species, as shown in Table 1.

**TABLE 1:** Morphological characteristic statistics of nine syringa species.

Species name	Leaf shape	Leaf base shape	Leaf color	Flowering period	Inflorescence shape	Petal type	Flower color
*Syringa oblata*	Cordate	Cordate	Dark green	Early	Panicle	Single	Light purple
*Syringa vulgaris*	Cordate	Cordate	Dark green	Early	Panicle	Double	White
*Syringa wolfii*	Elliptical	Cuneate	Light green	Mid	Dense panicle	Single	Light purple
*Syringa villosa*	Ovate	Cuneate	Light green	Mid	Dense panicle	Single	Light magenta
*Syringa josikaea*	Cordate	Rounded	Light green	Mid	Dense panicle	Single	Purple
*Syringa reticulata subsp. Pekinensis*	Elliptical	Rounded	Dark green	Cuneate	Panicle	Single	Light yellow
*Syringa reticulata subsp. Amurensis*	Elliptical	Rounded	Green	Cuneate	Loose panicle	Single	Light yellow
*Syringa pubescens subsp. Patula Palibin*	Ovate	Cuneate	Dark green	Mid	Loose panicle	Single	Magenta
*Syringa meyeri*	Ovate	Truncate	Green	Mid	Panicle	Single	Light magenta

In taxonomy, *Syringa* species are divided into two sections: *Sect. Syringa* and *Sect. Ligustrina. Sect. Syringa comprises Ser. Pinnatifoliae, Ser. Pubescentes (Schneid.) Lingelsh., Ser. Syringa,* and *Ser. Villosae (Schneid.) Rehd*, while *Sect. Ligustrina* consists of *Syringa pekinensis* and *Syringa reticulata*. The study found that species within the same taxonomic group exhibit more similar morphological characteristics. For instance, *Syringa oblata* and *Syringa vulgaris*, both belonging to *Ser. Syringa*, show similarities in leaf shape, leaf base shape, leaf color, lowering period, and Inflorescence shape. However, *Syringa vulgaris* exhibits a double petal morphology and a different color compared to *Syringa oblata*. Among *Syringa wolfii*, *Syringa villosa*, and *Syringa josikaea*, which are all classified under *Ser. Villosae (Schneid.) Rehd.*, similarities are observed in leaf color, lowering period, Inflorescence shape, and Petal type, while differences exist in leaf shape, leaf base shape, and flower color. *Syringa* pubescen*s subsp. Patula Palibin* and *Syringa meyeri*, both belonging to *Ser. Pubescentes (Schneid.) Lingelsh.*, share similar morphological characteristics in leaf shape, lowering period, and Petal type, but exhibit differences in leaf base shape, leaf color, Inflorescence shape, and flower color. Lastly, *Syringa reticulata subsp. Pekinensis* and *Syringa reticulata subsp. Amurensis*, which are both part of *Sect. Ligustrina*, show high similarity in eaf shape, leaf base shape, flowering period, Petal type, and flower color, with only slight differences in leaf color and Inflorescence shape.

### 2.2 Sequence characteristic analysis

The single and combined sequences of nine species of *Syringa* were compared and the sequence information is shown in Table 2. The length of in-dividual sequences varied from 225 bp (*ITS2*) to 510 bp (*psbA-trnH*). Among the sequences, *psbA-trnH* had the highest proportion of informative sites (55/480 bp), fol-lowed by *ITS2* (25/225 bp), *trnL-trnF* (12/351 bp), and *trnL* (8/510 bp). The combined sequences ranged in length from 576 bp (*ITS2* + *trnL-trnF*) to 1,566 bp (*ITS2* + *psbA-trnH* + *trnL-trnF* + *trnL*), with the number of informative sites for each combination being *ITS2*+*psbA-trnH* (54/705), *ITS2*+*psbA-trnH* + *trnL-trnF* (62/1,056), *psbA-trnH* + *trnL-trnF* (46/831), *ITS2*+*psbA-trnH* + *trnL* (56/1,215), *ITS2*+*trnL-trnF* (24/576), *ITS2*+*psbA-trnH* + *trnL-trnF* + *trnL* (64/1,566), *psbA-trnH* + *trnL* (40/990), *psbA-trnH* + *trnL-trnF* + *trnL* (48/1,341), *trnL-trnF* + *trnL* (24/861), *ITS2*+*trnL* (18/735), and *ITS2*+*trnL-trnF* + *trnL* (26/1,086).

**TABLE 2:** Statistics of sequence characteristics.

Sequence	Conserved sites	Variable sites	Parsimony-informative sites	Singleton sites	Total
*ITS2*	199	26	25	1	225
*psbA-trnH*	474	55	55	0	480
*trnL-trnF*	341	12	12	0	351
*trnL*	502	8	8	0	510
*ITS2*+*psbA-trnH*	628	80	54	26	705
*ITS2*+*trnL-trnF*	534	37	24	13	576
*ITS2*+*trnL*	702	32	18	14	735
*psbA-trnH* + *trnL-trnF*	764	67	46	21	831
*psbA-trnH* + *trnL*	932	62	40	22	990
*trnL-trnF* + *trnL*	836	42	24	18	861
*ITS2*+*psbA-trnH* + *trnL-trnF*	963	92	62	30	1,056
*ITS2*+*psbA-trnH* + *trnL*	1,131	87	56	31	1,215
*ITS2*+*trnL-trnF* + *trnL*	1,036	45	26	19	1,086
*psbA-trnH* + *trnL-trnF* + *trnL*	1,266	75	48	27	1,341
*ITS2*+*psbA-trnH* + *trnL-trnF* + *trnL*	1,465	100	64	36	1,566

### 2.3 Analysis of genetic distances and frequency distribution

The mean intraspecific and interspecific genetic distances for single and combined sequences were studied, as shown in Table 3. The interspecific distances ranged from 0.0044 to 0.0549, while the intraspecific distances ranged from 0.0004 to 0.0104. These distances can be utilized to assess the genetic variation of the sequences. Notably, *psbA-trnH* exhibited the highest genetic variation for both interspecific and intraspecific variations, the *trnL* intron showed the lowest interspecific genetic variation, and *trnL-trnF* had the lowest intraspecific genetic variation. In all sequences, interspecific distances were higher than intraspecific distances. The interspecific distances ranged from 0.0108 to 0.0678, and the intraspecific distances ranged from 0.0032 to 0.0126. The highest interspecific genetic variation was observed in the combined sequences *ITS2* + *psbA-trnH*, while the lowest interspecific and intraspecific genetic variation was found in *trnL-trnF* + *trnL*.

**TABLE 3:** Interspecific and intraspecific distance analysis of sequences based on the Kimura two-parameter model.

Sequences	Interspecific and intraspecific distance analysis		
	Average interspecific distances	Average intraspecific distance	Total mean genetic distance
*ITS2*	0.0399 ± 0.0119	0.0021 ± 0.0015	0.0367 ± 0.0069
*psbA-trnH*	0.0549 ± 0.0098	0.0104 ± 0.0029	0.0511 ± 0.0061
*trnL-trnF*	0.0096 ± 0.0050	0.0004 ± 0.0004	0.0089 ± 0.0029
*trnL*	0.0044 ± 0.0024	0.0008 ± 0.0006	0.0042 ± 0.0015
*ITS2*+*psbA-trnH*	0.0678 ± 0.0085	0.0086 ± 0.0023	0.0496 ± 0.0044
*ITS2*+*trnL-trnF*	0.0431 ± 0.0078	0.0084 ± 0.0025	0.0319 ± 0.0040
*ITS2*+*trnL*	0.0301 ± 0.0054	0.0049 ± 0.0016	0.0217 ± 0.0027
*psbA-trnH* + *trnL-trnF*	0.0439 ± 0.0067	0.0080 ± 0.0022	0.0357 ± 0.0036
*psbA-trnH* + *trnL*	0.0345 ± 0.0054	0.0085 ± 0.0021	0.0299 ± 0.0029
*trnL-trnF* + *trnL*	0.0108 ± 0.0031	0.0032 ± 0.0011	0.0088 ± 0.0016
*ITS2*+*psbA-trnH* + *trnL-trnF*	0.0588 ± 0.0063	0.0116 ± 0.0022	0.0447 ± 0.0033
*ITS2*+*psbA-trnH* + *trnL*	0.0440 ± 0.0053	0.0094 ± 0.0019	0.0351 ± 0.0027
*ITS2*+*trnL-trnF* + *trnL*	0.0283 ± 0.0046	0.0070 ± 0.0019	0.0211 ± 0.0023
*psbA-trnH* + *trnL-trnF* + *trnL*	0.0360 ± 0.0045	0.0126 ± 0.0022	0.0302 ± 0.0023
*ITS2*+*psbA-trnH* + *trnL-trnF* + *trnL*	0.0415 ± 0.0047	0.0101 ± 0.0019	0.0320 ± 0.0026

**FIGURE 1:**
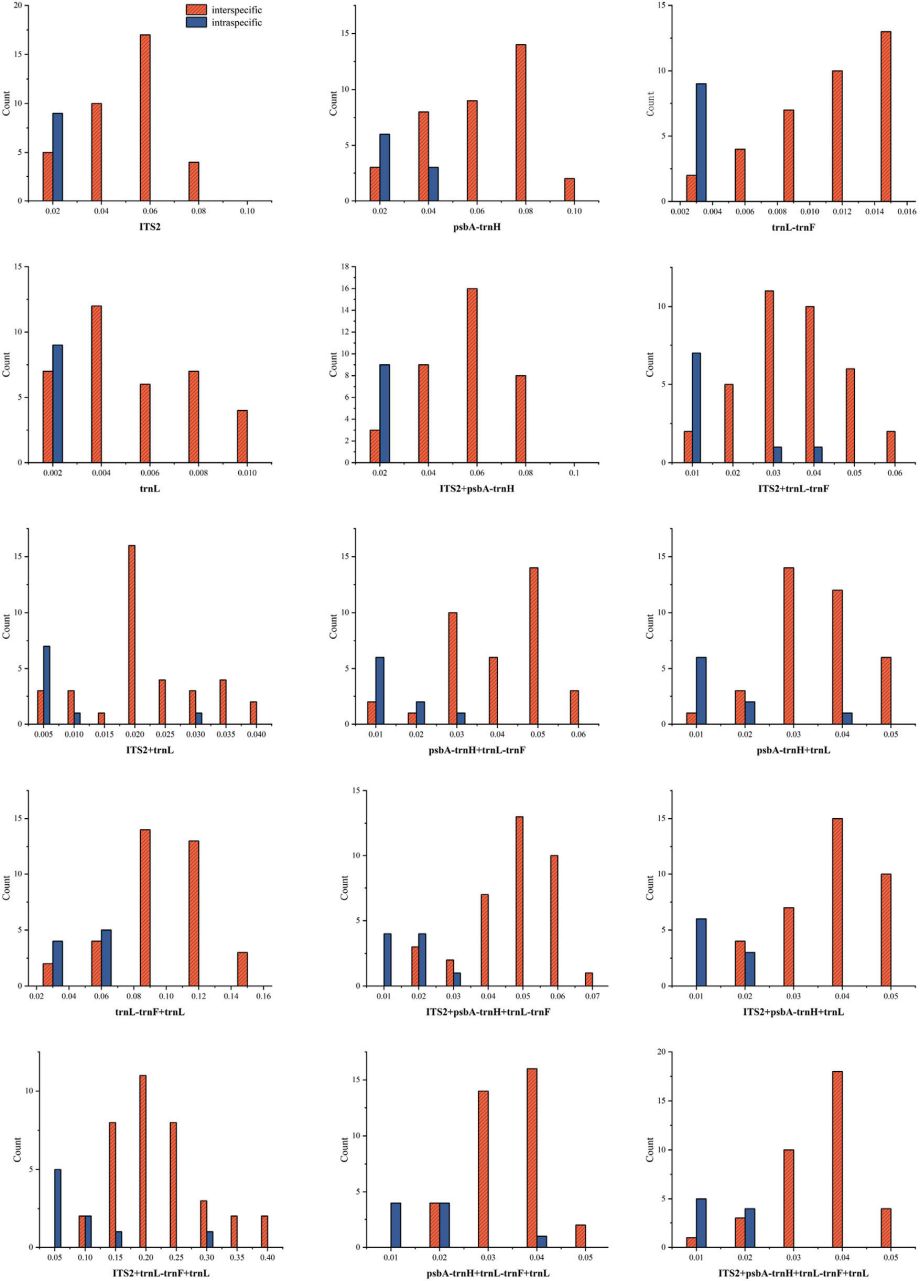
Distribution of interspecific and intraspecific distances of sequences (A: *ITS2*; B: *psbA- trnH*; C: *trnL-trnF*; D: *trnL*; E: *ITS2* + *psbA- trnH* ; F: *ITS2* + *trnL-trnF*; G: *ITS2* + *trnL*; H: *psbA- trnH + trnL-trnF*; I: *psbA- trnH + trnL*; J: *trnL-trnF + trnL*; K: *ITS2 + psbA- trnH + trnL-trnF*; L: *ITS2 + psbA- trnH + trnL*; M: *ITS2 + trnL-trnF + trnL*; N: *psbA-trnH + trnL-trnF + trnL*; O: *ITS2 + psbA-trnH + trnL-trnF + trnL*).

The frequency distribution of intraspecific and interspecific genetic distances is depicted in Figure 1. The results indicate that for single sequences, *ITS2*, *trnL*, and *trnL-trnF* showed relatively stable intraspecific variation, with overlapping distributions for both interspecific and intraspecific distances. For combined sequences, *ITS2* + *psbA-trnH* showed a stable trend in intraspecific variation, and all combined sequences exhibited overlapping intraspecific and interspecific distances.

### 2.4 Wilcoxon signed-rank test analysis

The interspecific distances of single and combined sequences were subjected to Wilcoxon Signed-Rank Test analysis, with results presented in Tables 4 and 5. The order of single sequences was *psbA-trnH* > *ITS2* > *trnL-trnF* > *trnL*. The order of combined sequences was *ITS2*+*psbA-trnH* > *ITS2*+*psbA-trnH* + *trnL-trnF* > *ITS2*+*psbA-trnH* + *trnL*, *psbA-trnH* + *trnL-trnF* > *ITS2*+*trnL-trnF*, *ITS2*+*psbA-trnH* + *trnL-trnF* + *trnL*, *psbA + trnH* + *trnL-trnF* + *trnL*, *psbA-trnH* + *trnL* > *ITS2*+*trnL*, *ITS2*+*trnL-trnF* + *trnL* > *trnL-trnF* + *trnL*. In the Wilcoxon Signed-Rank Test analysis, a p-value less than 0.05 is considered to indicate statistically significant with a meaningful difference between two sequences. Notably, comparisons could not be made between *ITS2*+*trnL-trnF* and *psbA-trnH* + *trnL*, *psbA-trnH* + *trnL-trnF* + *trnL*, and *ITS2*+*psbA-trnH* + *trnL-trnF* + *trnL* due to not statistically significant (p > 0.05). Additionally, comparisons could not be made between *ITS2*+*trnL* and *ITS2*+*trnL-trnF* + *trnL*, and between *ITS2*+*psbA-trnH* + *trnL* and *psbA-trnH* + *trnL-trnF* due to not statistically significant (p > 0.05). Furthermore, this study also tested the interspecific distances of single and combined sequences (Supplementary Table S1). The results indicate that the interspecific divergence of the single sequence *psbA-trnH* is higher than that of all combined sequences. The interspecific divergence of the single sequence *ITS2* is generally greater than that of the combined sequences. The interspecific divergence of the single sequences *trnL-trnF* and *trnL* is lower than that of the combined sequences.

**TABLE 4:** Wilcoxon signed-rank test for the interspecies distances of the single sequences.

W^+^	W^−^	Relative ranks		n	p	Results
*ITS2*	*psbA-trnH*	W^+^ = 0	W^−^ = 666	36	0.001	*ITS2* < *psbA-trnH*
*ITS2*	*trnL-trnF*	W^+^ = 666	W^−^ = 0	36	0.001	*ITS2* > *trnL-trnF*
*ITS2*	*trnL*	W^+^ = 666	W^−^ = 0	36	0.001	*ITS2* > *trnL*
*psbA-trnH*	*trnL-trnF*	W^+^ = 666	W^−^ = 0	36	0.001	*psbA-trnH* > *trnL-trnF*
*psbA-trnH*	*trnL*	W^+^ = 666	W^−^ = 0	36	0.001	*psbA-trnH* > *trnL*
*trnL-trnF*	*trnL*	W^+^ = 665	W^−^ = 1	36	0.001	*trnL-trnF* > *trnL*

**TABLE 5:** Wilcoxon signed-rank test for the interspecies distances of the combined sequences.

W^+^	W^−^	Relative ranks		n	p	Results
*ITS2*+*psbA-trnH*	*ITS2*+*trnL-trnF*	W^+^ = 665	W^−^ = 1	36	0.001	*ITS2*+*psbA-trnH* > *ITS2*+*trnL-trnF*
*ITS2*+*psbA-trnH*	*ITS2*+*trnL*	W^+^ = 666	W^−^ = 0	36	0.001	*ITS2*+*psbA-trnH* > *ITS2*+*trnL*
*ITS2*+*psbA-trnH*	*psbA-trnH* + *trnL-trnF*	W^+^ = 666	W^−^ = 0	36	0.001	*ITS2*+*psbA-trnH* > *psbA-trnH* + *trnL-trnF*
*ITS2*+*psbA-trnH*	*psbA-trnH* + *trnL*	W^+^ = 664	W^−^ = 2	36	0.001	*ITS2*+*psbA-trnH* > *psbA-trnH* + *trnL*
*ITS2*+*psbA-trnH*	*trnL-trnF* + *trnL*	W^+^ = 666	W^−^ = 0	36	0.001	*ITS2*+*psbA-trnH* > *trnL-trnF* + *trnL*
*ITS2*+*psbA-trnH*	*ITS2*+*psbA-trnH* + *trnL-trnF*	W^+^ = 595.5	W^−^ = 70.5	36	0.001	*ITS2*+*psbA-trnH* > *ITS2*+*psbA-trnH* + *trnL-trnF*
*ITS2*+*psbA-trnH*	*ITS2*+*psbA-trnH* + *trnL*	W^+^ = 654.5	W^−^ = 11.5	36	0.001	*ITS2*+*psbA-trnH* > *ITS2*+*psbA-trnH* + *trnL*
*ITS2*+*psbA-trnH*	*ITS2*+*trnL-trnF* + *trnL*	W^+^ = 660	W^−^ = 6	36	0.001	*ITS2*+*psbA-trnH* > *ITS2*+*trnL-trnF* + *trnL*
*ITS2*+*psbA-trnH*	*psbA-trnH* + *trnL-trnF* + *trnL*	W^+^ = 660	W^−^ = 6	36	0.001	*ITS2*+*psbA-trnH* > *psbA-trnH* + *trnL-trnF* + *trnL*
*ITS2*+*psbA-trnH*	*ITS2*+*psbA-trnH* + *trnL-trnF* + *trnL*	W^+^ = 661	W^−^ = 5	36	0.001	*ITS2*+*psbA-trnH* > *ITS2*+*psbA-trnH* + *trnL-trnF* + *trnL*
*ITS2*+*trnL-trnF*	*ITS2*+*trnL*	W^+^ = 630	W^−^ = 0	36	0.001	*ITS2*+*trnL-trnF* > *ITS2*+*trnL*
*ITS2*+*trnL-trnF*	*psbA-trnH* + *trnL-trnF*	W^+^ = 11	W^−^ = 655	36	0.001	*ITS2*+*trnL-trnF* < *psbA-trnH* + *trnL-trnF*
*ITS2*+*trnL-trnF*	*psbA-trnH* + *trnL*	W^+^ = 256	W^−^ = 410	36	0.226	*ITS2*+*trnL-trnF* < *psbA-trnH* + *trnL*
*ITS2*+*trnL-trnF*	*trnL-trnF* + *trnL*	W^+^ = 665	W^−^ = 1	36	0.001	*ITS2*+*trnL-trnF* > *trnL-trnF* + *trnL*
*ITS2*+*trnL-trnF*	*ITS2*+*psbA-trnH* + *trnL-trnF*	W^+^ = 0	W^−^ = 666	36	0.001	*ITS2*+*trnL-trnF* < *ITS2*+*psbA-trnH* + *trnL-trnF*
*ITS2*+*trnL-trnF*	*ITS2*+*psbA-trnH* + *trnL*	W^+^ = 67	W^−^ = 563	36	0.001	*ITS2*+*trnL-trnF* < *ITS2*+*psbA-trnH* + *trnL*
*ITS2*+*trnL-trnF*	*ITS2*+*trnL-trnF* + *trnL*	W^+^ = 617	W^−^ = 49	36	0.001	*ITS2*+*trnL-trnF* > *ITS2*+*trnL-trnF* + *trnL*
*ITS2*+*trnL-trnF*	*psbA-trnH* + *trnL-trnF* + *trnL*	W^+^ = 366.5	W^−^ = 299.5	36	0.599	*ITS2*+*trnL-trnF* > *psbA-trnH* + *trnL-trnF* + *trnL*
*ITS2*+*trnL-trnF*	*ITS2*+*psbA-trnH* + *trnL-trnF* + *trnL*	W^+^ = 277	W^−^ = 353	36	0.534	*ITS2*+*trnL-trnF* < *ITS2*+*psbA-trnH* + *trnL-trnF* + *trnL*
*ITS2*+*trnL*	*psbA-trnH* + *trnL-trnF*	W^+^ = 0	W^−^ = 666	36	0.001	*ITS2*+*trnL* < *psbA-trnH* + *trnL-trnF*
*ITS2*+*trnL*	*psbA-trnH* + *trnL*	W^+^ = 0	W^−^ = 666	36	0.001	*ITS2*+*trnL* < *psbA-trnH* + *trnL*
*ITS2*+*trnL*	*trnL-trnF* + *trnL*	W^+^ = 658	W^−^ = 8	36	0.001	*ITS2*+*trnL* > *trnL-trnF* + *trnL*
*ITS2*+*trnL*	*ITS2*+*psbA-trnH* + *trnL-trnF*	W^+^ = 0	W^−^ = 666	36	0.001	*ITS2*+*trnL* < *ITS2*+*psbA-trnH* + *trnL-trnF*
*ITS2*+*trnL*	*ITS2*+*psbA-trnH* + *trnL*	W^+^ = 0	W^−^ = 666	36	0.001	*ITS2*+*trnL* < *ITS2*+*psbA-trnH* + *trnL-trnF*
*ITS2*+*trnL*	*ITS2*+*trnL-trnF* + *trnL*	W^+^ = 309	W^−^ = 357	36	0.706	*ITS2*+*trnL* < *ITS2*+*trnL-trnF* + *trnL*
*ITS2*+*trnL*	*psbA-trnH* + *trnL-trnF* + *trnL*	W^+^ = 0	W^−^ = 666	36	0.001	*ITS2*+*trnL* < *psbA-trnH* + *trnL-trnF* + *trnL*
*ITS2*+*trnL*	*ITS2*+*psbA-trnH* + *trnL-trnF* + *trnL*	W^+^ = 0	W^−^ = 666	36	0.001	*ITS2*+*trnL* < *ITS2*+*psbA-trnH* + *trnL-trnF* + *trnL*
*psbA-trnH* + *trnL-trnF*	*psbA-trnH* + *trnL*	W^+^ = 629	W^−^ = 37	36	0.001	*psbA-trnH* + *trnL-trnF* > *psbA-trnH* + *trnL*
*psbA-trnH* + *trnL-trnF*	*trnL-trnF* + *trnL*	W^+^ = 666	W^−^ = 0	36	0.001	*psbA-trnH* + *trnL-trnF* > *trnL-trnF* + *trnL*
*psbA-trnH* + *trnL-trnF*	*ITS2*+*psbA-trnH* + *trnL-trnF*	W^+^ = 0	W^−^ = 666	36	0.001	*psbA-trnH* + *trnL-trnF* < *ITS2*+*psbA-trnH* + *trnL-trnF*
*psbA-trnH* + *trnL-trnF*	*ITS2*+*psbA-trnH* + *trnL*	W^+^ = 395.5	W^−^ = 270.5	36	0.326	*psbA-trnH* + *trnL-trnF* > *ITS2*+*psbA-trnH* + *trnL*
*psbA-trnH* + *trnL-trnF*	*ITS2*+*trnL-trnF* + *trnL*	W^+^ = 645	W^−^ = 21	36	0.001	*psbA-trnH* + *trnL-trnF* > *ITS2*+*trnL-trnF* + *trnL*
*psbA-trnH* + *trnL-trnF*	*psbA-trnH* + *trnL-trnF* + *trnL*	W^+^ = 593	W^−^ = 73	36	0.001	*psbA-trnH* + *trnL-trnF* > *psbA-trnH* + *trnL-trnF* + *trnL*
*psbA-trnH* + *trnL-trnF*	*ITS2*+*psbA-trnH* + *trnL-trnF* + *trnL*	W^+^ = 558	W^−^ = 108	36	0.001	*psbA-trnH* + *trnL-trnF* > *ITS2*+*psbA-trnH* + *trnL-trnF* + *trnL*
*psbA-trnH* + *trnL*	*trnL-trnF* + *trnL*	W^+^ = 666	W^−^ = 0	36	0.001	*psbA-trnH* + *trnL* > *trnL-trnF* + *trnL*
*psbA-trnH* + *trnL*	*ITS2*+*psbA-trnH* + *trnL-trnF*	W^+^ = 0	W^−^ = 666	36	0.001	*psbA-trnH* + *trnL* < *ITS2*+*psbA-trnH* + *trnL-trnF*
*psbA-trnH* + *trnL*	*ITS2*+*psbA-trnH* + *trnL*	W^+^ = 0	W^−^ = 630	36	0.001	*psbA-trnH* + *trnL* < *ITS2*+*psbA-trnH* + *trnL*
*psbA-trnH* + *trnL*	*ITS2*+*trnL-trnF* + *trnL*	W^+^ = 638	W^−^ = 28	36	0.001	*psbA-trnH* + *trnL* > *ITS2*+*trnL-trnF* + *trnL*
*psbA-trnH* + *trnL*	*psbA-trnH* + *trnL-trnF* + *trnL*	W^+^ = 490	W^−^ = 176	36	0.014	*psbA-trnH* + *trnL* > *psbA-trnH* + *trnL-trnF* + *trnL*
*psbA-trnH* + *trnL*	*ITS2*+*psbA-trnH* + *trnL-trnF* + *trnL*	W^+^ = 331	W^−^ = 335	36	0.975	*psbA-trnH* + *trnL* < *ITS2*+*psbA-trnH* + *trnL-trnF* + *trnL*
*trnL-trnF* + *trnL*	*ITS2*+*psbA-trnH* + *trnL-trnF*	W^+^ = 0	W^−^ = 666	36	0.001	*trnL-trnF* + *trnL* < *ITS2*+*psbA-trnH* + *trnL-trnF*
*trnL-trnF* + *trnL*	*ITS2*+*psbA-trnH* + *trnL*	W^+^ = 0	W^−^ = 666	36	0.001	*trnL-trnF* + *trnL* < *ITS2*+*psbA-trnH* + *trnL*
*trnL-trnF* + *trnL*	*ITS2*+*trnL-trnF* + *trnL*	W^+^ = 1	W^−^ = 665	36	0.001	*trnL-trnF* + *trnL* < *ITS2*+*trnL-trnF* + *trnL*
*trnL-trnF* + *trnL*	*psbA-trnH* + *trnL-trnF* + *trnL*	W^+^ = 0	W^−^ = 666	36	0.001	*trnL-trnF* + *trnL* < *psbA-trnH* + *trnL-trnF* + *trnL*
*trnL-trnF* + *trnL*	*ITS2*+*psbA-trnH* + *trnL-trnF* + *trnL*	W^+^ = 0	W^−^ = 666	36	0.001	*trnL-trnF* + *trnL* < *ITS2*+*psbA-trnH* + *trnL-trnF* + *trnL*
*ITS2*+*psbA-trnH* + *trnL-trnF*	*ITS2*+*psbA-trnH* + *trnL*	W^+^ = 664	W^−^ = 2	36	0.001	*ITS2*+*psbA-trnH* + *trnL-trnF* > *ITS2*+*psbA-trnH* + *trnL*
*ITS2*+*psbA-trnH* + *trnL-trnF*	*ITS2*+*trnL-trnF* + *trnL*	W^+^ = 663	W^−^ = 3	36	0.001	*ITS2*+*psbA-trnH* + *trnL-trnF* > *ITS2*+*trnL-trnF* + *trnL*
*ITS2*+*psbA-trnH* + *trnL-trnF*	*psbA-trnH* + *trnL-trnF* + *trnL*	W^+^ = 666	W^−^ = 0	36	0.001	*ITS2*+*psbA-trnH* + *trnL-trnF* > *psbA-trnH* + *trnL-trnF* + *trnL*
*ITS2*+*psbA-trnH* + *trnL-trnF*	*ITS2*+*psbA-trnH* + *trnL-trnF* + *trnL*	W^+^ = 666	W^−^ = 0	36	0.001	*ITS2*+*psbA-trnH* + *trnL-trnF* > *ITS2*+*psbA-trnH* + *trnL-trnF* + *trnL*
*ITS2*+*psbA-trnH* + *trnL*	*ITS2*+*trnL-trnF* + *trnL*	W^+^ = 659.5	W^−^ = 6.5	36	0.001	*ITS2*+*psbA-trnH* + *trnL* > *ITS2*+*trnL-trnF* + *trnL*
*ITS2*+*psbA-trnH* + *trnL*	*psbA-trnH* + *trnL-trnF* + *trnL*	W^+^ = 660	W^−^ = 6	36	0.001	*ITS2*+*psbA-trnH* + *trnL* > *psbA-trnH* + *trnL-trnF* + *trnL*
*ITS2*+*psbA-trnH* + *trnL*	*ITS2*+*psbA-trnH* + *trnL-trnF* + *trnL*	W^+^ = 666	W^−^ = 0	36	0.001	*ITS2*+*psbA-trnH* + *trnL* > *ITS2*+*psbA-trnH* + *trnL-trnF* + *trnL*
*ITS2*+*trnL-trnF* + *trnL*	*psbA-trnH* + *trnL-trnF* + *trnL*	W^+^ = 29	W^−^ = 637	36	0.001	*ITS2*+*trnL-trnF* + *trnL* < *psbA-trnH* + *trnL-trnF* + *trnL*
*ITS2*+*trnL-trnF* + *trnL*	*ITS2*+*psbA-trnH* + *trnL-trnF* + *trnL*	W^+^ = 20	W^−^ = 646	36	0.001	*ITS2*+*trnL-trnF* + *trnL* < *ITS2*+*psbA-trnH* + *trnL-trnF* + *trnL*
*psbA-trnH* + *trnL-trnF* + *trnL*	*ITS2*+*psbA-trnH* + *trnL-trnF* + *trnL*	W^+^ = 47.5	W^−^ = 582.5	36	0.001	*psbA-trnH* + *trnL-trnF* + *trnL* < *ITS2*+*psbA-trnH* + *trnL-trnF* + *trnL*

### 2.5 Analysis based on BLAST searches

**FIGURE 2:**
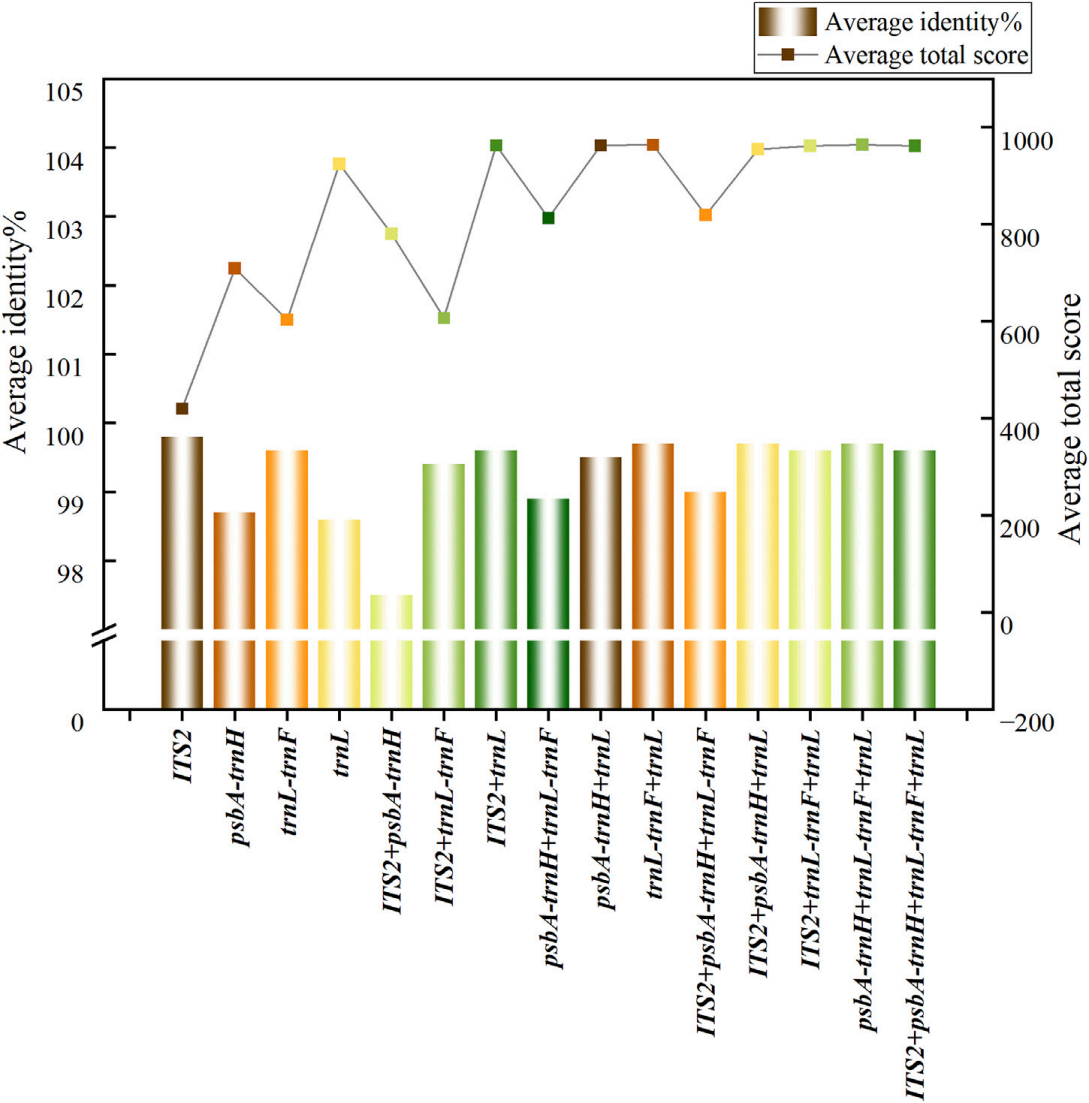
Trends of average score and average recognition rate in BLAST analysis.

The average scores, alignment rates and recognition rates of single and combined sequences of 9 *Syringa* species retrieved by BLAST from NCBI are shown in Supplementary Table S2. The average scores and recognition rates of different sequences are shown in Figure 2. Among the single sequences, the *trnL* sequence had the highest score, while the *ITS2* sequence had the lowest. In the combined sequences, the *psbA-trnH* + *trnL-trnF* + *trnL* sequence had the highest score, and the *ITS2*+*trnL-trnF* sequence had the lowest. For single sequences, the *ITS2* had the highest average identification rate at 99.80%, while the *trnL* had the lowest at 98.61%. The average identification rates for the *ITS2* and *trnL-trnF* sequences ranged from 99% to 100%, and for the *psbA-trnH* and *trnL* sequences, they ranged from 98% to 99%. Among the combined sequences *ITS2*+*psbA-trnH* + *trnL* had the highest identification rates at 99.70%, while *ITS2+psbA-trnH* had the lowest at 97.51%. The average identification rates for the sequences *ITS2*+*trnL-trnF*, *ITS2*+*trnL*, *psbA-trnH* + *trnL*, *trnL-trnF* + *trnL*, *ITS2*+*psbA-trnH* + *trnL*, *ITS2*+*trnL-trnF* + *trnL*, *psbA-trnH* + *trnL-trnF* + *trnL*, and *ITS2*+*psbA-trnH* + *trnL-trnF* + *trnL* ranged from 99% to 100%, while the average identification rates for the *ITS2*+*psbA-trnH*, *ITS2*+*psbA-trnH* + *trnL-trnF* and *psbA-trnH* + *trnL-trnF* sequences ranged from 97% to 99%.

### 2.6 Sequence analysis based on characters

The identification rates and logical formulas for each sequence based on the BLOG algorithm are presented in Table 6. For the nine *Syringa* species, the single sequence *psbA-trnH* and all combined sequences achieved a correct classification rate of 100%. Among the single sequences, the *trnL-trnF* sequence revealed specific base positions for different species: *S*. *reticulata subsp. Amurensis* has G at position 88, T at position 189, and A at position 192; *S*. *reticulata subsp. Pekinensis* has T at position 88; *S*. *pubescens subsp. patula Palibin* has A at position 192 and G at position 292; *S*. *meyeri* has G at position 189 and A at position 292; *S*. *villosa* has A at position 79, C at position 192, and A at position 292; *S*. *wolfii* has C at position 79; *S*. *josikaea* has C at position 192 and G at position 292; whereas *S*. *oblata* and *S*. *vulgaris* did not have corresponding positions to distinguish them. In the *trnL* sequence, *S. oblata* has T at position 10; *S. vulgaris* has T at position 10 and A at position 198; *S. reticulata subsp. Amurensis* has G at position 88 and T at position 189, and A at position 192; *S. reticulata subsp. Pekinensis* has A at position 278; *S. pubescens subsp. patula Palibin* has A at position 12 and G at position 278; *S. meyeri* has G at position 12, G at position 198, and G at position 278; *S. wolfii* has A at position 12 and C at position 385; while *S. villosa* and *S*. *josikaea* did not have corresponding positions to distinguish them. Among the combined sequences, all nine *Syringa* species had corresponding base positions for differentiation.

**TABLE 6:** Character-based approach for species identification.

Sequences				Formula								
	cc	wc	nc	*S. oblata*	*S. vulgaris*	*S. reticulata subsp*. *Amurensis*	*S. reticulata subsp*. *Pekinensis*	*S. pubescens subsp*. Patula Palibin	*S. meyeri*	*S. villosa*	*S. wolfii*	*S. josikaea*
*ITS2*	50	40	10	168 = A	24 = C 39 = T 168 = A	39 = C 49 = T	31 = A	24 = T 39 = C	24 = C 31 = T	39 = T 49 = C 86 = C	86 = T	24 = T 49 = T
*psbA-trnH*	100	0	0	449 = A 472 = A	362 = T	271 = G 362 = G	362 = C	84 = G	4 = T	141 = G 381 = A	5 = C	390 = A 532 = G
*trnL-trnF*	77.78	0	22.22	-	-	88 = G 189 = T 192 = A	88 = T	192 = A 292 = G	189 = G 292 = A	79 = A 192 = C 292 = A	79 = C	192 = C 292 = G
*trnL*	77.78	0	22.22	10 = T	10 = T 198 = A	12 = G 278 = G	278 = A	12 = A 278 = G	12 = G 198 = G 278 = G	-	12 = A 395 = C	-
*ITS2*+*psbA-trnH*	100	0	0	438 = G 677 = A	567 = T	476 = G 595 = A	567 = C	293 = G	293 = A	438 = A 595 = G	86 = T	595 = A 737 = G
*ITS2*+*trnL-trnF*	100	0	0	170 = A	51 = T 154 = T 170 = A	41 = C 51 = T	41 = C 51 = C 154 = C	24 = T 154 = T	24 = C 51 = C	41 = T 51 = C 88 = C	88 = T	24 = T 51 = T
*ITS2*+*trnL*	100	0	0	170 = A	24 = C 41 = T 170 = A	41 = C 51 = T	24 = C 240 = A	24 = T 41 = C	41 = C 51 = C	41 = T 51 = C 240 = G	41 = T 240 = A	24 = T 51 = T
*psbA-trnH* + *trnL-trnF*	100	0	0	561 = A	358 = T	550 = C	566 = C	84 = G	4 = T	438 = G 559 = T	5 = C	551 = A
*psbA-trnH* + *trnL*	100	0	0	543 = T	358 = T	551 = C	358 = C	84 = G	4 = T	120 = C 551 = G	5 = C	541 = C 551 = G
*trnL-trnF* + *trnL*	100	0	0	352 = T	352 = T 540 = A	246 = A 354 = G 540 = G	618 = T	292 = G 354 = A	189 = G 354 = G	292 = A 354 = G	79 = C	292 = G 737 = C
*ITS2*+*psbA-trnH* + *trnL-trnF*	100	0	0	785 = A 787 = T	585 = T	786 = C	785 = T	311 = G	24 = C 49 = C	364 = G 786 = G	779 = T	784 = G 1121 = G
*ITS2*+*psbA-trnH* + *trnL*	100	0	0	776 = A	584 = T	467 = T	781 = C	310 = G	230 = T	816 = C	231 = C	746 = G 766 = A
*ITS2*+*trnL-trnF* + *trnL*	100	0	0	168 = A	49 = T 152 = T	39 = C 49 = T	39 = C 49 = C 152 = C	24 = T 152 = T	24 = C 49 = C 152 = T	39 = T 49 = C 86 = C	86 = T	24 = T 49 = T
*psbA-trnH* + *trnL-trnF* + *trnL*	100	0	0	543 = T	358 = T	551 = C	358 = C	84 = G	4 = T	460 = G 540 = G	78 = T	549 = G 879 = G
*ITS2*+*psbA-trnH* + *trnL-trnF* + *trnL*	100	0	0	766 = T	581 = T	774 = C	581 = C	310 = G	230 = T	774 = G 781 = A	86 = T	763 = A 1100 = G

### 2.7 Tree-based analysis

#### 2.7.1 NJ tree analysis of a single sequence

**FIGURE 3:**
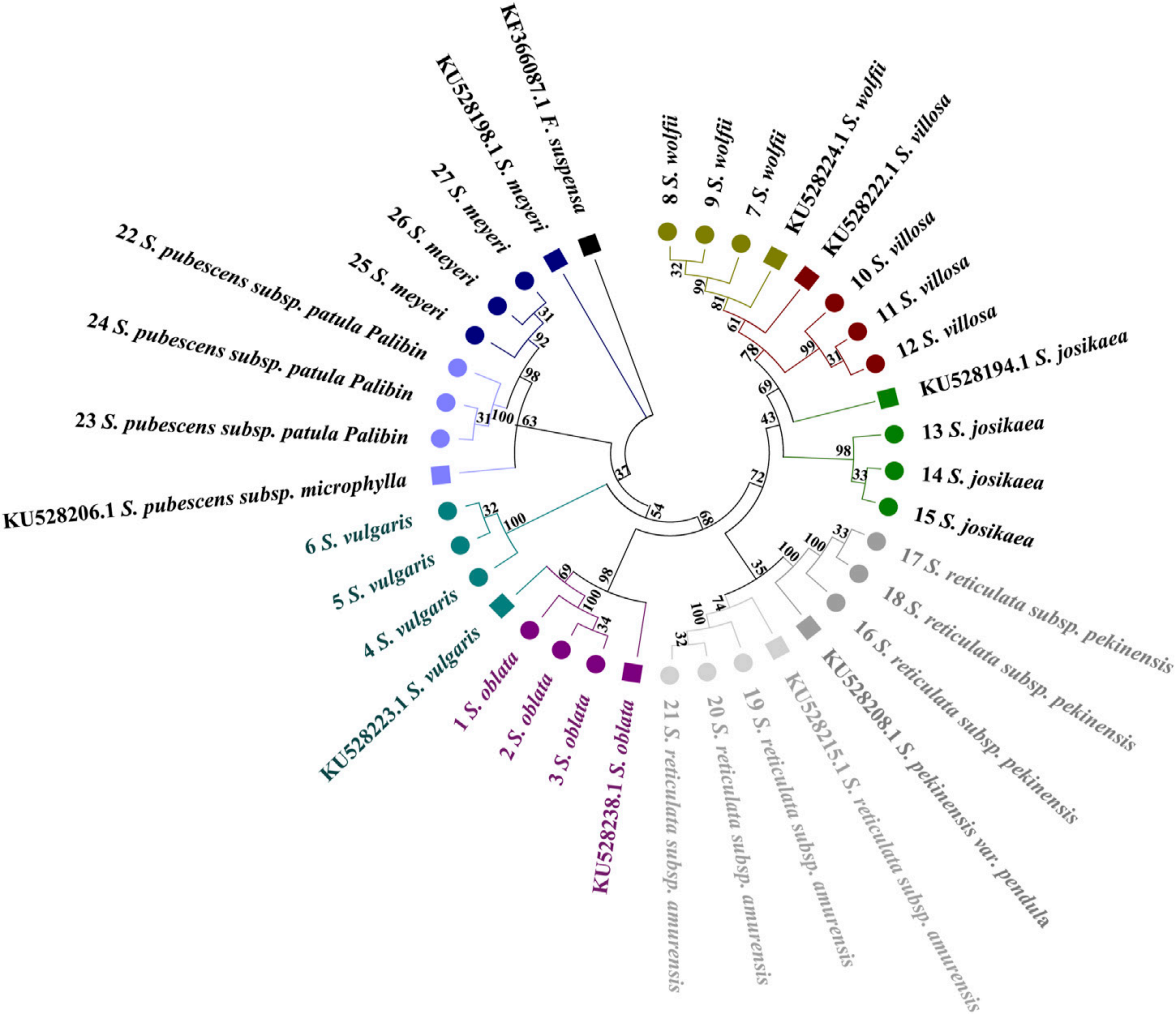
Phylogenetic tree based on the *ITS2* alignment matrix of 27 samples from 9 *Syringa* species. The *F*. *suspensa ITS2* sequence (GenBank accession number: MG219753.1) was used as an outgroup to root the tree. Taxa are color-coded at the species level for easy discrimination of each species. *Syringa* species analyzed in this study are marked with circles. Numbers in the species labels correspond to sample ID (Table 6). Reference sequences obtained from GenBank are marked with squares and accession numbers in the taxon labels.

The *Forsythia suspensa* ITS2 sequence was used as an outgroup to root the tree. In the single sequence NJ tree analysis of the *ITS2* region, a broad distribution pattern is observed (Figure 3), with specimens from the evolutionary branches formed by the species pairs *(S. oblata* and *S. vulgaris*), (*S. villosa* and *S. wolfii*), and (*S. pubescens subsp. Patula Palibin* and *S. meyeri*) showing clear clustering. However, the *ITS2* sequence differences between the two species in each of these pairs are minimal, preventing further sub-clustering. From the figure, it is evident that *S. oblata* and *S. vulgaris* form the basal branches, followed by a larger branch composed of two sub-branches: one grouping *S. villosa*, *S. wolfii*, and *S. josikaea*; the other sub-branch divides into two smaller branches, with *S. pubescens subsp. Paulat palibin* and *S. meyeri* forming one branch; and *S. reticulata subsp. Pekinensis* and *S. reticulata subsp. Amurensis* forming the other. The tree diagram clearly shows that *S. reticulata subsp. Pekinensis*, *S. reticulata subsp. Amurensis*, and *S. josikaea* can be distinctly clustered and form separate sub-clusters.

**FIGURE 4:**
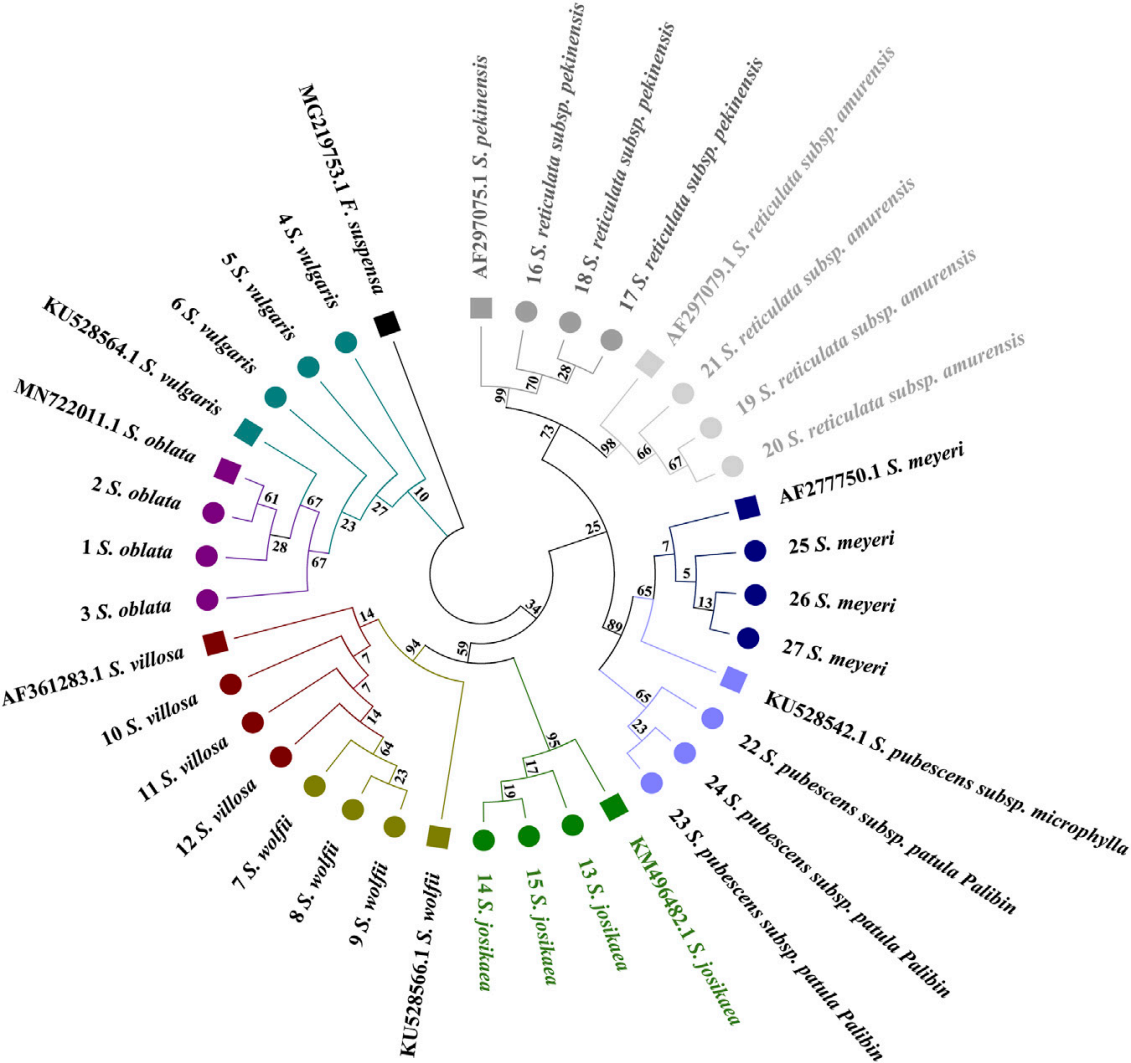
Phylogenetic tree generated from the *psbA-trnH* alignment matrix of 27 samples from 9 *Syringa* species. The *F*. *suspensa psbA-trnH* sequence (GenBank accession number: KF366087.1) was used as an outgroup to root the tree. Taxa are color-coded at the species level for easy discrimination of each species. *Syringa* species analyzed in this study are marked with circles. Numbers in the species labels correspond to sample ID (Table 6). Reference sequences obtained from GenBank are marked with squares and accession numbers in the taxon labels.

Distinct distribution patterns from those observed with the *ITS2* barcode are evident in the *psbA-trnH* tree (Figure 4). The phylogenetic tree is primarily divided into two main sections. *Syringa meyeri* and *S. pubescens subsp. Patula Palibin* form a basal clade, followed by the *S. vulgaris* clade. The last major branch consists of two sub-branches: *S. oblata* and a branch comprising *S. reticulata subsp. Amurensis*, *S. reticulata subsp. Pekinensis*, *S. josikaea*, *S. villosa*, and *S. wolfii*. High sequence similarity among *S*. *josikaea*, *S. villosa*, and *S. wolfii* presents challenges in further sub-clustering. Unlike the analysis of *ITS2* sequences, *S*. *oblata* and *S. vulgaris* do not form a single clade, with the reference sequence of *S. vulgaris* being clustered within *S. oblata*. However, it is clear from the tree that species specimens of *S. reticulata subsp. Pekinensis*, *S. reticulata subsp. Amurensis*, *S. oblata*, and *S. vulgaris* can be distinctly clustered and form separate sub-clusters.

**FIGURE 5:**
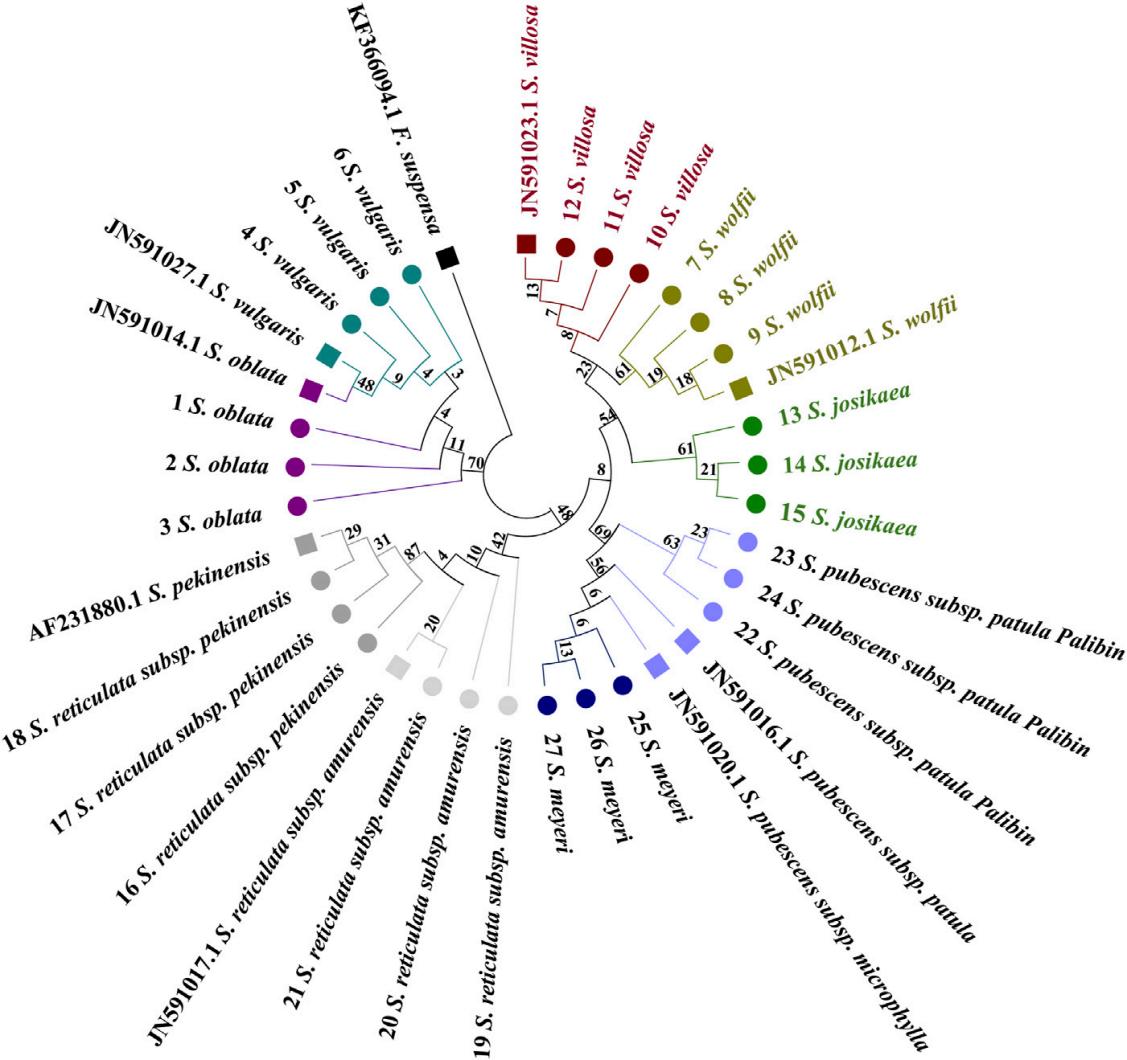
Phylogenetic tree generated from the *trnL-trnF* alignment matrix of 27 samples from 9 *Syringa* species. The *F*. *suspensa trnL-trnF* sequence (GenBank accession number: KF366094.1) was used as an outgroup to root the tree. Taxa are color-coded at the species level for easy discrimination of each species. *Syringa* species analyzed in this study are marked with circles. Numbers in the species labels correspond to sample ID (Table 6). Reference sequences obtained from GenBank are marked with squares and accession numbers in the taxon labels.

A distribution pattern similar to that observed with the *ITS2* barcode is clearly discernible in the *trnL-trnF* tree (Figure 5). Specimens from the evolutionary branches formed by *S. oblata* and *S. vulgaris*, *S. reticulata subsp. Pekinensis* and *S. reticulata subsp. Amurensis*, and *S. pubescens subsp. Patula Palibin* and *S. meyeri* are noticeably clustered. However, the *trnL-trnF* sequence differences between the two species in these three pairs are minimal, preventing further sub-clustering. The tree illustrates that *S. oblata* and *S. reticulata subsp. Pekinensis* form a basal branch, followed by a larger branch composed of two sub-branches, one consisting of *S. reticulata subsp. Pekinensis* and *S. reticulata subsp. Amurensis*, and the other divided into three branches, namely, *S. meyeri* and *S. pubescens subsp. Patula Palibin*, *S. josikaea* as a separate branch, and a branch composed of *S. villosa* and *S. wolfii*. The reference sequence of *S. pubescens subsp. Patula Palibin* and the specimens of *S. meyeri* cluster together. However, it is clear from the tree that specimens of *S. josikaea*, *S. wolfii*, and *S. villosa* can be distinctly clustered and form separate sub-clusters.

**FIGURE 6:**
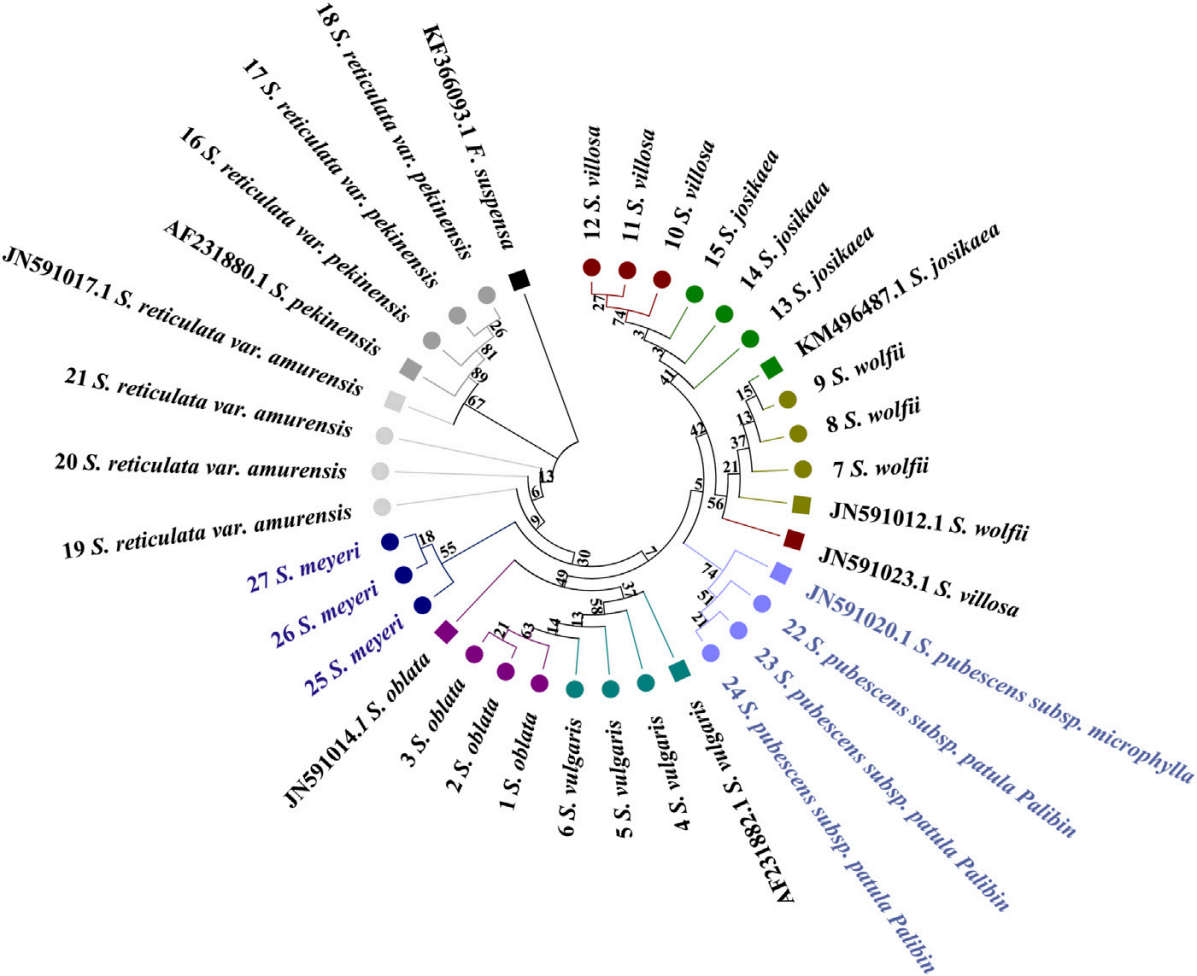
Phylogenetic tree generated from the *trnL* alignment matrix of 27 samples from 9 *Syringa* species. The *F*. *suspensa trnL* sequence (GenBank accession number: KF366093.1) was used as an outgroup to root the tree. Taxa are color-coded at the species level for easy discrimination of each species. *Syringa* species analyzed in this study are marked with circles. Numbers in the species labels correspond to sample ID (Table 6). Reference sequences obtained from GenBank are marked with squares and accession numbers in the taxon labels.

The *trnL* tree does not clearly exhibit a broad distribution pattern (Figure 6). The evolutionary tree is primarily divided into two sections. It can be observed that the sequences of *S. reticulata subsp. Pekinensis* and the reference sequence of *S. reticulata subsp. Amurensis* are positioned at the basal position, with the sample sequences of *S. reticulata subsp. Amurensis* following. Subsequently, a branch emerges, which splits into two smaller branches, one being *S. meyeri*, and the other branch further divides into two branches, one consisting of *S. oblata* and *S. vulgaris* forming a single branch, and the other branch splits again into two branches, one being *S. pubescens subsp. Patula Palibin*, and the other branch further divides into two branches, one branch being *S. wolfii*, and the other branch being *S. villosa* and *S. josikaea*. The reference sequence of *S*. *reticulata subsp. Amurensis* clusters with *S. reticulata subsp. Pekinensis*, the reference sequences of *S. villosa* and *S. josikaea* cluster with *S. wolfii*, and the reference sequence of *S. oblata* clusters with *S. vulgaris*, still presenting issues with high sequence similarity preventing sub-clustering. However, it is clear from the tree that the species specimens of *S. pubescens subsp. Patula Palibin* and *S. meyeri* can be distinctly clustered and form separate sub-clusters.

The results indicate that single sequences are unable to cluster all nine species of *Syringa* into a single clade on the phylogenetic tree. Instead, they can only group two or three species of *Syringa* together, with relatively high bootstrap values.

#### 2.7.2 Combined sequence NJ tree analysis

**FIGURE 7:**
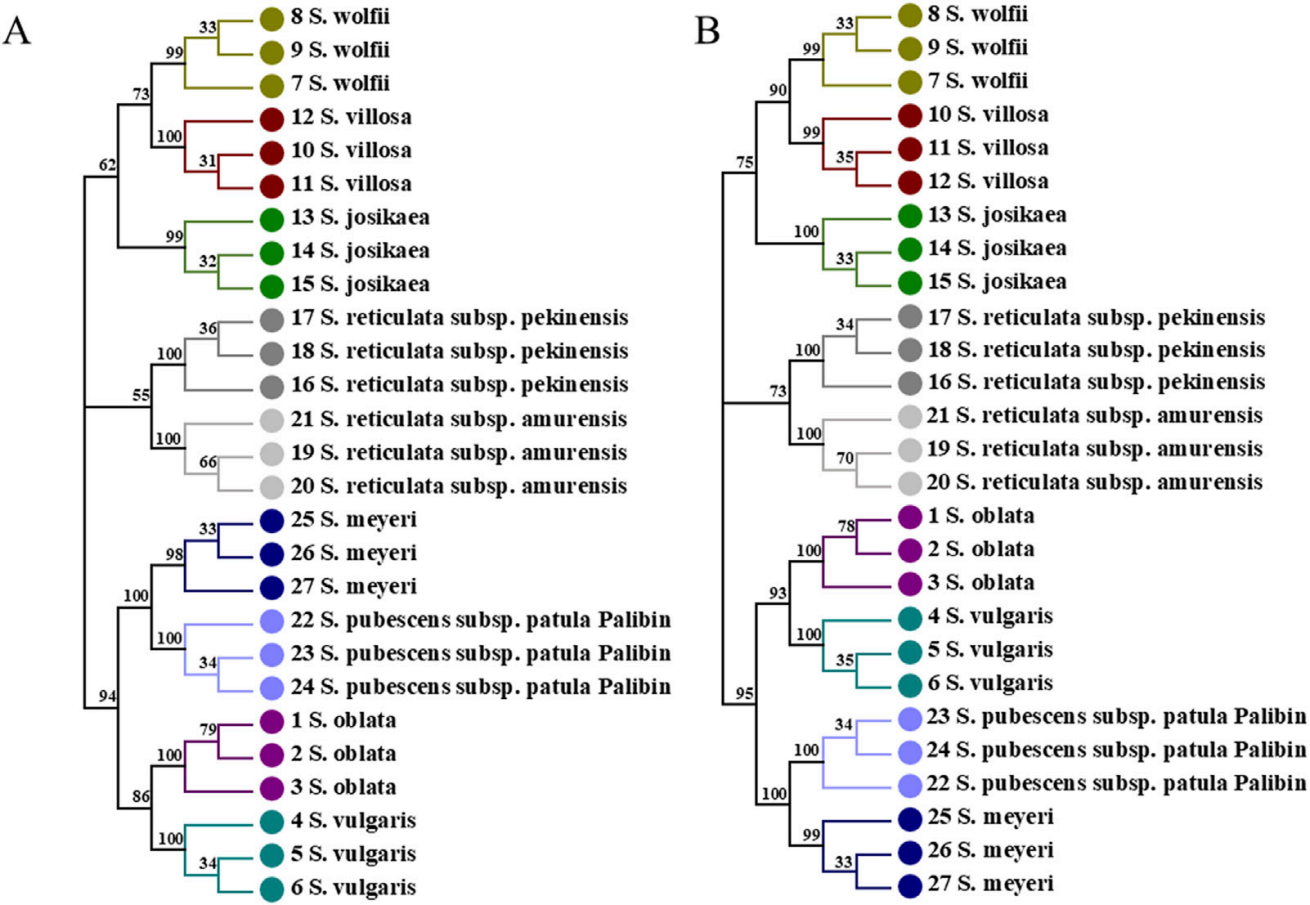
NJ trees constructed based on *ITS2+psbA-trnH + trnL*, *ITS2+psbA-trnH + trnL-trnF + trnL* [**(A)**: *ITS2+psbA-trnH + trnL;* **(B)** *ITS2+psbA-trnH + trnL-trnF + trnL*].

**FIGURE 8:**
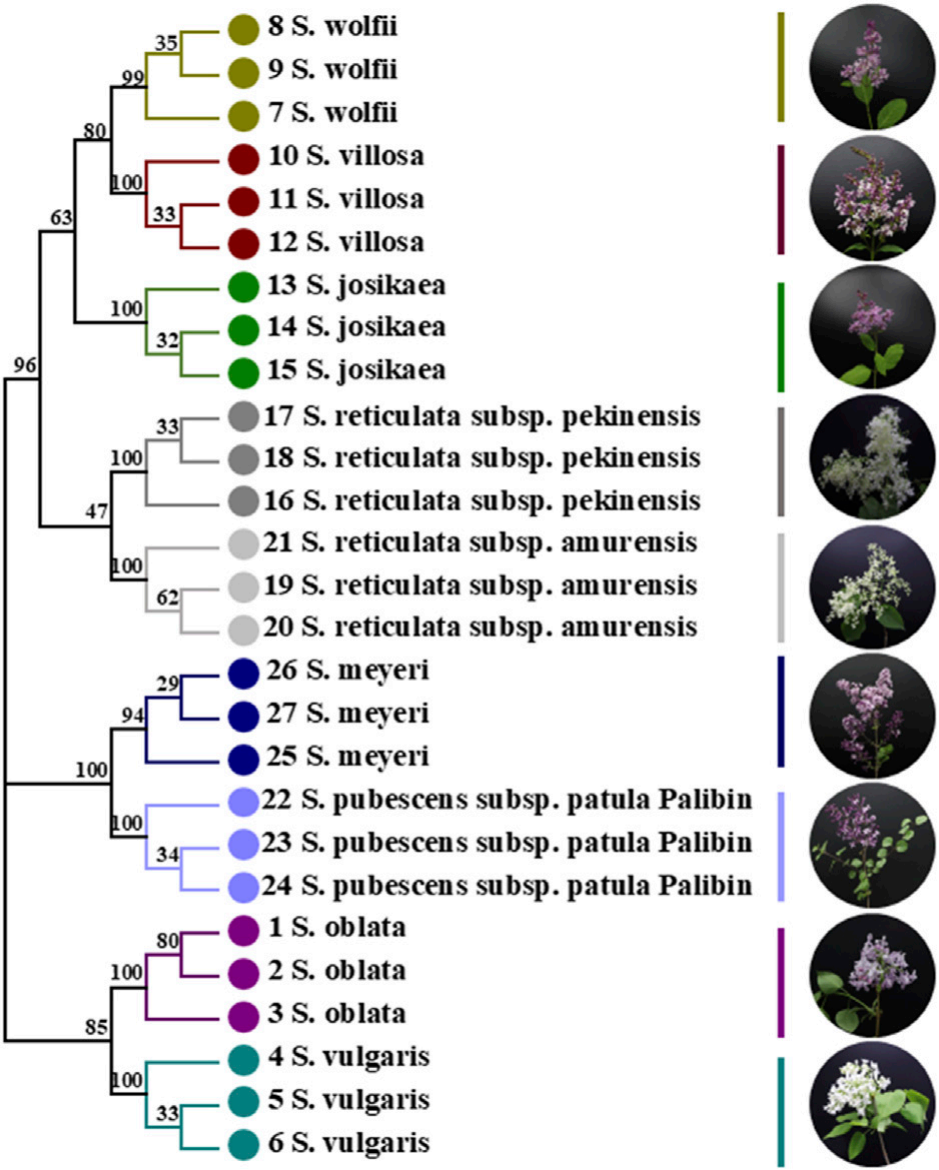
NJ trees constructed based on *ITS2+psbA-trnH + trnL-trnF*.

In the combined sequences, the NJ trees constructed with*ITS2*+*psbA-trnH* + *trnL*, and *ITS2*+*psbA-trnH* + *trnL-trnF* + *trnL* and *ITS2*+*psbA-trnH* + *trnL-trnF* align with the phylogenetic analysis model for *Syringa* species and are capable of distinguishing the nine *Syringa* species, with high support rates for individual sub-clustering (Figures 7A, B, 8). The identification success rates for the three combined sequences, *ITS2+psbA-trnH + trnL-trnF*, *ITS2+psbA-trnH + trnL*, and *ITS2+psbA-trnH + trnL-trnF + trnL*, were 93.6%, 70%, and 81%, respectively. Among these, the combination of *ITS2+psbA-trnH + trnL-trnF* exhibited the highest identification success rate. Among other sequences, the NJ tree constructed with *ITS2*+*psbA-trnH* sequences clusters *S. wolfii* and *S. villosa* together, failing to distinguish between these two species (Supplementary Figure S1). In the NJ tree constructed with *ITS2*+*trnL-trnF* sequences, *S. wolfii* and *S. villosa* cluster together, and *S. oblata* and *S. vulgaris* also cluster together, preventing the distinction between these two pairs of species (Supplementary Figure S2). In the NJ trees constructed with *ITS2*+*trnL* and *ITS2*+*trnL-trnF* + *trnL* sequences, *S. oblata* and *S. vulgaris* cluster together, failing to distinguish between *S. oblata* and *S. vulgaris* (Supplementary Figures S3, S7). In the NJ tree constructed with *trnL-trnF* + *trnL* sequences, *S. oblata* and *S. vulgaris* cluster together, and *S. reticulata subsp. Amurensis* as a single species fails to cluster, making it unclear to distinguish these three species (Supplementary Figure S6). The NJ trees constructed with *psbA-trnH* + *trnL-trnF, psbA-trnH* + *trnL*, and *psbA-trnH* + *trnL-trnF* + *trnL* sequences do not conform to the phylogenetic analysis model for *Syringa* species but can complete individual sub-clustering (Supplementary Figures S4, S5, S8). Therefore, these three combined sequences cannot be used to identify the nine *Syringa* species.

The results demonstrated that individual sequences were inadequate for distinguishing among the nine species of the genus *Syringa*. Among the combinations of sequences, three specific combinations were effective in differentiating the nine species of *Syringa*. Notably, the sequence combination of I*TS2+psbA-trnH + trnL-trnF* achieved the highest identification rate, reaching up to 93.6%.

## 3 Discussions

### 3.1 Comparative analysis of DNA barcode analysis methods

Current DNA barcode analysis methods primarily include sequence feature analysis, genetic distance analysis, BLAST search analysis in NCBI, and evolutionary tree construction analysis (Zhang et al., 2015). The genetic distance analysis and phylogenetic tree construction methods have been applied in the identification of plants in the genus *Syringa* to assess whether the combination of *psbA-trnH* and *trnC-petN* sequences can serve as a DNA barcode for *Syringa* plants (Yao et al., 2022). In this study, the analysis methods were expanded to include BLAST analysis and BLOG method analysis, and the effectiveness of these four analysis methods in the selection of DNA barcodes for nine species of *Syringa* was evaluated.

For genetic distances, intraspecific and interspecific genetic distances are generally assessed, with the ideal DNA barcode characterized by small intraspecific distances and a significant difference from interspecific distances (Hao et al., 2012). Wilcoxon signed-rank tests can further analyze interspecific genetic distances (Kim and Kim, 2003; Kasuya, 2010). DNA barcodes, combined with the distribution of genetic distances among sequences and the results of Wilcoxon signed-rank tests, indicate that the *ITS2*+*psbA-trnH* sequence exhibits stable intraspecific variation and significant interspecific variation. However, this method could not evaluate the identification effect of barcodes. BLAST is a commonly used method to compare sequence similarities in the NCBI database, comparing identification effects based on ratios and scores. The results indicate that the BLAST method exhibits a high identification rate, with all sequences achieving identification rates between 97% and 100%. Among the single sequences, *ITS2* has the highest identification rate at 99.80%. Among the combined sequences, the *ITS2* + *psbA-trnH* + *trnL* sequence has the highest identification rate at 99.70%. It shows that the BLAST-based method has a high identification rate at the genus level. Character-based analysis methods identify species through different base substitutions at specific positions in the sequence, typically using BLOG analysis software. This method was applied in this study to select DNA barcodes for *Syringa* genus species. The BLOG algorithm was used to analyze the DNA barcodes of the nine *Syringa* genus plants, with an accurate identification rate of 50% for *ITS2*, and 77.78% for *trnL-trnF* and *trnL*, while the remaining individual and combined sequences could accurately identify these nine species.

The construction of phylogenetic trees using UPGMA, NJ, and MP methods serves as a basis for DNA barcode identification assessment (Liu et al., 2015). The Neighbor-Joining (NJ) method is relatively accurate for constructing phylogenetic trees when the evolutionary distances are short and the number of informative sites is limited in short sequences (Li and Gao, 2009). The results of this study show that NJ trees constructed with *ITS2*+*psbA-trnH* + *trnL-trn* conform to the phylogenetic analysis model of *Syringa* genus plants and the results obtained in this study are in alignment with those reported by preceding scholars (He M., 2007). In summary, distance-based methods and BLAST methods cannot directly assess the identification effects of DNA barcodes, but character-based and tree-based methods can. In this study, the construction of phylogenetic trees showed the best identification effects.

### 3.2 Comparison of discrimination ability of single and combined sequences

Based on current research on the structure of the nuclear and chloroplast genomes, it is difficult to find a universal DNA barcode suitable for all plants. Since Kress proposed the idea of sequence combinations, more studies have proven that combined sequences have higher species discrimination ability than single sequences (Kress et al., 2005). In the study of *Syringa* genus species, two barcode sequences and their combinations were analyzed for 33 species, revealing that combined sequences have higher identification capabilities than any single sequence (Yao et al., 2022).

This study analyzed four single sequences and 11 combined sequences of *Syringa* genus plants. The results of distance-based analysis indicated that, except for *ITS2* and *psbA-trnH*, the average interspecific distance of combined sequences was higher than that of other single sequences, supported by the results of Wilcoxon signed-rank tests. However, the genetic distance distribution showed that intraspecific variation was more stable for single sequences compared to combined sequences, with all sequences having overlapping regions. BLAST-based analysis results indicated that combined sequences had higher scores than single sequences, but both single and combined sequences had high identification success rates. Feature-based analysis showed that only one single sequence could accurately identify *Syringa* genus plants, while all sequence combinations could achieve this goal, indicating a significant improvement in identification ability compared to single sequences. In the NJ tree analysis, none of the four single sequences could cluster the nine *Syringa* species separately. However, among the combined sequences, six were able to cluster the nine plant species. Yet, only the three NJ trees constructed using *ITS2*+*psbA-trnH + trnL-trnL*, *ITS2+psbA-trnH + trnL*, and *ITS2+psbA-trnH + trnL-trnF + trnL* enabling the separate clustering of these nine species. Ultimately, the sequence combination of ITS2+psbA-trnH + trnL-trnF was selected for its highest accuracy in identification. Therefore, the results of this study indicate that combined sequences have higher identification capabilities for these nine *Syringa* genus plants compared to single sequences. However, not all combined sequences can accurately identify these nine species, which is also related to the choice of analysis method.

### 3.3 Morphological discussion and DNA barcode selection of nine *Syringa* species

The results obtained in this study regarding the morphological traits of *Syringa* species in the northeast region are consistent with those collected by previous researchers in their cladistic analysis of the *Syringa* genus based on morphological traits. However, the morphological clustering analysis does not support the traditional classification results (He N., 2007). Traditional morphological markers are significantly influenced by the developmental stage of plants and environmental factors, making it difficult to effectively distinguish species with very similar morphologies.

Currently, molecular marker techniques are also frequently employed in species classification and identification due to their characteristics of being rapid, accurate, and objective. Specifically, polymorphisms such as AFLP, SSR, and ISSR are identified through the amplification of DNA fragments and the detection of changes in DNA length (Varma and Shrivastava, 2018; Gyorgy et al., 2020; Kocsisne et al., 2020; Yang et al., 2020). Study have shown that ISSR molecular marker techniques can be used for the identification of plants in the genus *Syringa* (Yao et al., 2021). The results indicate that there is an overlapping phenomenon in the clustering of species between the *Ser. Pubescentes (Schneid.) Lingelsh* and the *Ser. Villosae (Schneid.) Rehd*. This finding is consistent with the results of the AFLP analysis on the phylogenetic relationships of plants in the genus *Syringa* (Gao et al., 2011). Therefore, neither of these markers can accurately distinguish between these two groups. With the rapid development of sequencing technologies, the method of species identification using DNA sequences has been recognized as reliable and accurate. DNA barcoding technology can accurately identify species through variation sites in marker sequences. In previous studies, different researchers have utilized nuclear genomic sequences such as *ITS* and *ETS*, as well as chloroplast genomic sequences like *psbA-trnH*, *trnL-trnF*, and *trnC-petN* for the identification and phylogenetic analysis of plants in the genus *Syringa*. This study employed four analytical methods, namely, distance-based methods, BLAST-based methods, character-based methods, and tree-based methods, to evaluate whether the *ITS2* + *psbA-trnH* + *trnL-trnF* sequences could serve as a barcode for nine species of *Syringa*. The study incorporated the *trnL* intron and chloroplast genome sequences, which had not been used in previous *Syringa* research, with the aim of screening new DNA barcodes suitable for differentiating these nine tree species from the sequences.

From the perspective of genetic distance distribution results, the inter-specific variation of the *ITS2* + *psbA-trnH* + *trnL-trnF* sequence overlaps with the intra-specific variation, and the inter-specific variation distance is relatively large. The analysis based on BLAST indicates that this sequence can achieve a species-level identification rate of 98.97%. The analysis based on sequence characteristics also shows that the sequence has an accuracy of 100% for the nine tree species. In the NJ tree constructed based on *ITS2* + *psbA-trnH* + *trnL-trnF*, the nine species of *Syringa* can be clustered into three different clades, among which *Sect. Ligustrina* and *Ser. Villosae (Schneid.) Rehd*. Cluster together, and *Ser. Syringa* and *Ser. Pubescentes (Schneid.) Lingelsh* each form a separate clade. The research results are similar to those of the NJ tree results from the identification of *Syringa* based on chloroplast genomes. However, in the NJ phylogenetic tree constructed using the combined sequences of *psbA-trnH* and *trnC-petN*, *Ser. Villosae (Schneid.) Rehd.* Clusters separately and is closer to the root. The results are also similar to the NJ phylogenetic tree constructed using the *trnL-trnF* single sequence in the molecular systematics study of *Syringa* in the Northeast region, where Ser. *Syringa* is closer to the root (He M., 2007). In the NJ phylogenetic tree constructed using the combined sequences of *psbA-trnH* and *trnC-petN*, *Ser. Villosae (Schneid.) Rehd*. Clusters separately and is closer to the root (Yao et al., 2022). The presentation of these results may be related to the selection of sequences. The experimental results obtained from multiple evaluation methods demonstrate that the *ITS2* + *psbA-trnH* + *trnL-trnF* sequence has strong discriminatory power, providing strong support for the conclusion that it can serve as a DNA barcode for these nine tree species. In addition to *ITS2* + *psbA-trnH* + *trnL-trnF*, in the combined sequence research, *ITS2* + *psbA-trnH* + *trnL* and *ITS2* + *psbA-trnH* + *trnL-trnF* + trnL can also distinguish the nine species of *Syringa*, but the identification rate is lower than that of the *ITS2* + *psbA-trnH* + *trnL-trnF* sequence. In conclusion, it is recommended to use the *ITS2* + *psbA-trnH* + *trnL-trnF* sequence as the DNA barcode for the identification of the nine species of *Syringa*.

Through the combined analysis of morphological methods and DNA barcoding technology, the results of the barcode *ITS2* + *psbA-trnH* + *trnL-trnF* in the identification of nine species of *Syringa* were verified. The phylogenetic tree of the DNA barcode *ITS2 + psbA-trnH + trnL-trnF* shows that *S. oblata* and *S. vulgaris*, which have similar morphological features such as leaf shape, leaf base shape, leaf color, lowering period, and Inflorescence shape, cluster within *Ser. Syringa*. *Syringa wolfii*, *S. villosa*, and *S. josikaea*, which have similar features such as leaf color, lowering period, Inflorescence shape, and Petal type, cluster within *Ser. Villosae (Schneid.) Rehd*. *Syringa oblata* and *S. vulgaris*, which have similar features such as leaf shape, lowering period, and Petal type, cluster within *Ser. Pubescentes (Schneid.) Lingelsh*. *Syringa reticulata subsp. Pekinensis* and *S. reticulata subsp. Amurensis*, which have similar features such as leaf shape, leaf base shape, flowering period, and Petal type, cluster within *Sect. Ligustrina*. This is consistent with previous morphological and taxonomic research results. Excluding the group division, *Sect. Ligustrina* is included in *Sect. Syringa*. Therefore, currently, the use of morphological feature analysis and single or combined barcode fragments can only be applied and identified within a small range of higher plants (at the family, genus, and species levels), and the results of phylogenetic analysis may be incorrect or contradictory to traditional taxonomy. It is evident that the screening of traditional plant barcodes still has a long way to go.

## 4 Materials and methods

### 4.1 Plant materials and DNA extraction

**FIGURE 9:**
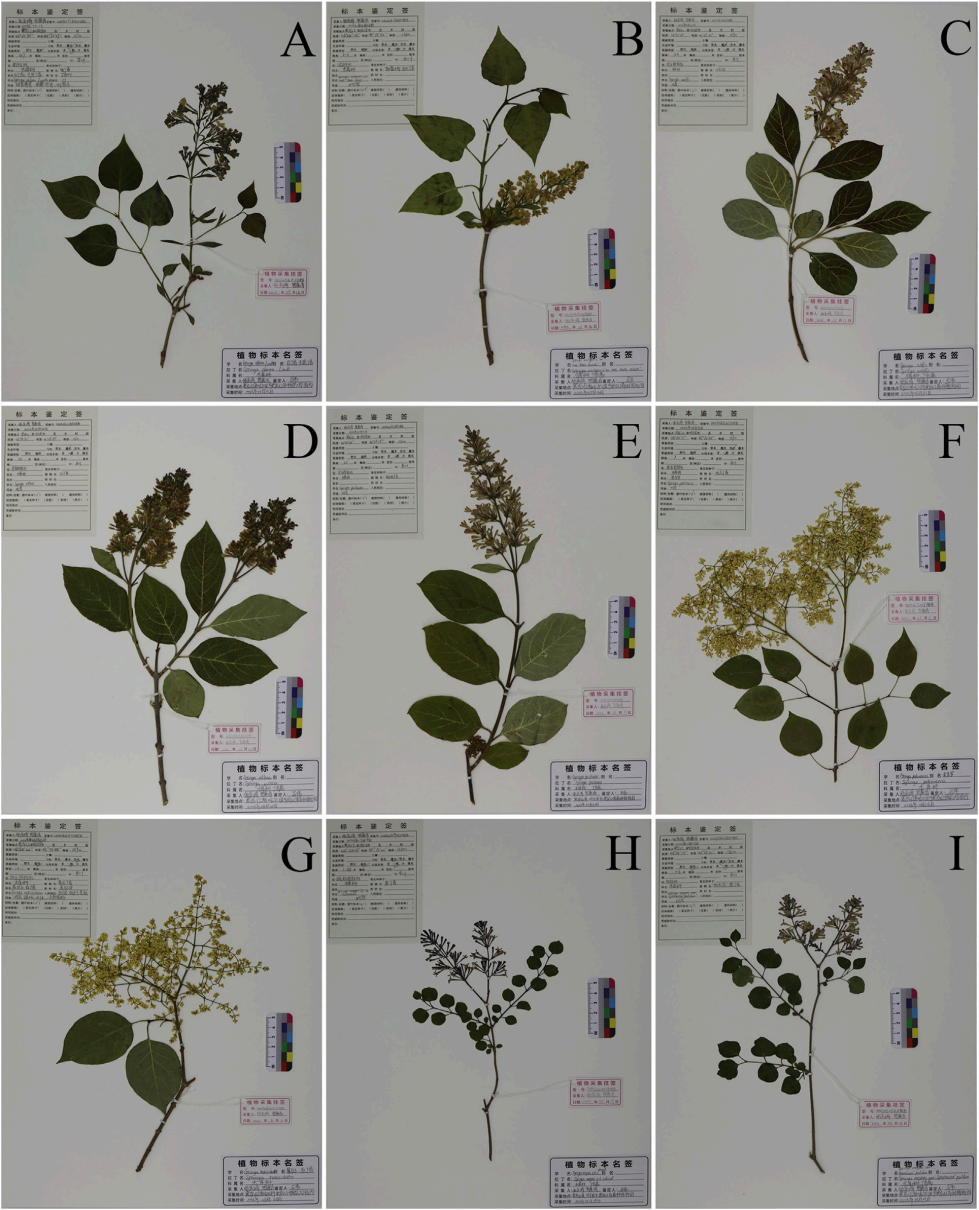
Nine species of *Syringa* L. Herbarium specimens. **(A)** *S.oblata* (Serial Number:20230513X1HRB); **(B)** *S.vulgaris* (Serial Number:20230516Z5HRB); **(C)** *S.wolfii* (Serial Number:20230531Z7HRB); **(D)** *S.villosa* (Serial Number:20230602Z6HRB); **(E)** *S.josikaea* (Serial Number:20230531Z8HRB); **(F)** *S.reticulata subsp. Pekinensis* (Serial Number:20230616X17HRB); **(G)** *S.reticulata subsp. Amurensis* (Serial Number:20230606X11HRB); **(H)** *S.pubescens subsp. Patula Palibin* (Serial Number:20230519X10HRB); **(I)** *S.meyeri* (Serial Number:20230525Z1HRB).

The seeds of 27 specimens of nine different species of *syringa* were provided by the Botanical Garden of Heilongjiang University of Chinese Medicine Pharmacy Botanical Garden and The Tree Specimen Garden of the Heilongjiang Forest Botanical Garden. All plant samples were identified by researcher Ma Wei, Department of Chinese Medicine Resources, Heilongjiang University of Chinese Medicine. Species names and source information are shown in Table 7. The wax leaf specimens of nine species of *Syringa* are shown in Figure 9.

**TABLE 7:** Information of the plant samples.

Species name	Sample	Longitude	Latitude	Collection locations	Sample types
*S.oblata*	1–3	126°63′93″E	45°72′27″N	Heilongjiang University of Chinese Medicine	Wild
*S.vulgaris*	4–6	126°64′26″E	45°69′62″N	Heilongjiang Forest Botanical Garden tree specimen garden	Cultivated
*S.wolfii*	7–9	126°64′29″E	45°69′63″N	Heilongjiang Forest Botanical Garden tree specimen garden	Cultivated
*S.villosa*	10–12	126°64′32″E	45°69′59″N	Heilongjiang Forest Botanical Garden tree specimen garden	Cultivated
*S.josikaea*	13–15	126°64′26″E	45°69′59″N	Heilongjiang Forest Botanical Garden tree specimen garden	Cultivated
*S.reticulata subsp. Pekinensis*	16–18	126°64′11″E	45°72′45″N	Heilongjiang University of Chinese Medicine	Wild
*S.reticulata subsp. Amurensis*	19–21	126°64′02″E	45°72′48″N	Heilongjiang University of Chinese Medicine	Wild
*S.pubescens subsp. Patula Palibin*	22–24	126°64′29″E	45°72′31″N	Heilongjiang University of Chinese Medicine	Wild
*S.meyeri*	25–27	126°64′25″E	45°69′61″N	Heilongjiang Forest Botanical Garden tree specimen garden	Cultivated

For each sample, three mature fresh leaves of the whole plant in different orientations were taken in a pre-cooled mortar, ground and crushed with liquid nitrogen, and the DNA of the samples was extracted using the Plant Genomic DNA Extraction Kit (TIANGEN), and the extracted DNA was stored at −20°C.

### 4.2 PCR amplification and sequence analysis

PCR amplification was performed to a final volume of 25 µL in an eppendorf research thermocycler (Eppendorf AG 22331, Hamburg, Germany). The reaction mixture contained 2 µM genomic DNA, 1 µM forward and reverse primers, and 8.5 mM ddH_2_O, 12.5 mM 2 × MEGA Fast Taq Master Mix (Msunflowers, China). The primer sequences used for the DNA barcoding analyses are shown in Supplementary Table S4. PCR cycles consisted of an initial denaturation step for 5 min at 94°C, followed by 30 cycles of denaturation (30 s at 94°C), annealing (30 s at 58°C) and elongation (1 min at 72°C), a final elongation at 72°C for 10 min. The PCR products were sequenced in two directions with magnetic bead method in an automated ABI 3730 sequencer (PE Applied Biosystems). Sequence ambiguities were manually corrected using the Sequencing analysis software version 5.2 (Carlsbad, California, United States of America).

### 4.3 Data analysis

MEGA7 software was used to perform multiple alignment of sequences, and the basic information of each sequence was calculated after manually adjusting the sequence. In addition, in this study, *ITS2* + *psbA-trnH*, *ITS2* + *trnL-trnF*, *ITS2* + *trnL*, *psbA-trnH* + *trnL-trnF*, *psbA-trnH* + *trnL*, *psbA-trnH* + *trnL*, *trnL-trnF* + *trnL*, *ITS2* + *psbA-trnH* + *trnL-trnF*, *ITS2* + *psbA-trnH* + *trnL*, *psbA-trnH* + *trnL-trnF* + *trnL*, *ITS2* + *psbA-trnH* + *trnL-trnF* + *trnL*, *ITS2* + *psbA-trnH* + *trnL-trnF* + *trnL* were selected as candidate DNA barcodes for further identification and analysis. The GenBank accession numbers of each sequence are shown in Supplementary Table S3. Kimura two-parameter (K2P) model in MEGA seven software was used to calculate genetic distance. Three parameters, interspecific, intraspecific and mean genetic distance, were used to evaluate the results. The distribution of genetic variation was observed by intraspecific and interspecific genetic distances. DNA barcode sequences should show independent and non-overlapping distributions of genetic variation in intraspecific and interspecific samples. The method of Wilcoxon signed rank test was used to verify the significance of the difference among the species by using IBM SPSS Statistics 27 software. In recognition ability, BLAST, character method and evolutionary tree method were selected to evaluate each sequence (Bosmali et al., 2022). Blast search was performed in NCBI database, and the most similar uploaded sequences of the same species were selected for statistical analysis, and the recognition ability of each sequence was evaluated. Blog 2.0 is based on sequence feature analysis, using classification rules to analyze the features of base sites (Weitschek et al., 2013). In this study, Blog 2.0 software was used to evaluate the discrimination rate of different sequences, and the logic rules were obtained. MEGA7 is used to build adjacent (NJ) trees (Ross et al., 2008), and the Bootstrap support option is set to 1,000 random addition replicates to determine the branch’s statistical support. When all individuals of a species can congregate in a single clade, the species is considered to have been successfully identified.

## 5 Conclusion

In this study, we described the morphological characteristics of nine *Syringa* species. Employing DNA barcoding techniques, four different methods were utilized to evaluate the identification capabilities of four single sequences and eleven combined sequences for the nine *Syringa* tree species. Across all methods, the sequences demonstrated the best identification performance when analyzed using the NJ tree approach. Moreover, compared to single sequences, combined sequences showed a notable enhancement in identification capabilities when employing the character-based method. Experimental results indicated that single sequences could only identify 2-3 out of the nine *Syringa* genus plants, whereas the combined sequence analyses, specifically *ITS2*+*psbA-trnH* + *trnL-trnF* accurately distinguished all nine species of *Syringa* genus plants using the four evaluation methods, exhibiting excellent discrimination and identification capabilities. The study ultimately selected the combination of *ITS2+psbA-trnH + trnL-trnF* as the optimal DNA barcode for the identification of nine species within the genus *Syringa*.

## Data Availability

The datasets presented in this study can be found in online repositories. The names of the repository/repositories and accession number(s) can be found in the article/Supplementary Material.
