# Supplementary material for: Morphological description and DNA barcoding research of nine Syringa species
Source: Front Genet. 2025 Feb 26;16:1544062. doi: 10.3389/fgene.2025.1544062 (PMC11897579; doi:10.3389/fgene.2025.1544062)
Supplement: Supplementary file 1 [file Table1.docx]

Supplementary Material

| **Table S1.** Wilcoxon signed-rank test for interspecies distances between the single and combined sequences | | | | | | | | |  |
| --- | --- | --- | --- | --- | --- | --- | --- | --- | --- |
| W^＋^ | W^－^ | Relative Ranks |  | n | | p | results | |  |
| *ITS2* | *ITS2+psbA-trnH* | W^＋^=3 | W^－^=663 | 36 | 0.001 | | | *ITS2＜ITS2+psbA-trnH* | |
| *ITS2* | *ITS2+trnL-trnF* | W^＋^=597.5 | W^－^=32.5 | 36 | 0.001 | | | *ITS2＞ITS2+trnL-trnF* | |
| *ITS2* | *ITS2+trnL* | W^＋^=663.5 | W^－^=2.5 | 36 | 0.001 | | | *ITS2＞ITS2+trnL* | |
| *ITS2* | *psbA-trnH+trnL-trnF* | W^＋^=575.5 | W^－^=90.5 | 36 | 0.001 | | | *ITS2＞psbA-trnH+trnL-trnF* | |
| *ITS2* | *psbA-trnH+trnL* | W^＋^=622 | W^－^=44 | 36 | 0.001 | | | *ITS2＞psbA-trnH+trnL* | |
| *ITS2* | *trnL-trnF+trnL* | W^＋^=663 | W^－^=3 | 36 | 0.001 | | | *ITS2＞trnL-trnF+trnL* | |
| *ITS2* | *ITS2+psbA-trnH+trnL-trnF* | W^＋^=136.5 | W^－^=493.5 | 36 | 0.003 | | | *ITS2＜ITS2+psbA-trnH+trnL-trnF* | |
| *ITS2* | *ITS2+psbA-trnH+trnL* | W^＋^=549 | W^－^=117 | 36 | 0.001 | | | *ITS2＞ITS2+psbA-trnH+trnL* | |
| *ITS2* | *ITS2+trnL-trnF+trnL* | W^＋^=658 | W^－^=8 | 36 | 0.001 | | | *ITS2＞ITS2+trnL-trnF+trnL* | |
| *ITS2* | *psbA-trnH+trnL-trnF+trnL* | W^＋^=611 | W^－^=55 | 36 | 0.001 | | | *ITS2＞psbA-trnH+trnL-trnF+trnL* | |
| *ITS2* | *ITS2+psbA-trnH+trnL-trnF+trnL* | W^＋^=570 | W^－^=60 | 36 | 0.001 | | | *ITS2＞ITS2+psbA-trnH+trnL-trnF+trnL* | |
| *psbA-trnH* | *ITS2+psbA-trnH* | W^＋^=666 | W^－^=0 | 36 | 0.001 | | | *psbA-trnH＞ITS2+psbA-trnH* | |
| *psbA-trnH* | *ITS2+trnL-trnF* | W^＋^=666 | W^－^=0 | 36 | 0.001 | | | *psbA-trnH＞ITS2+trnL-trnF* | |
| *psbA-trnH* | *ITS2+trnL* | W^＋^=666 | W^－^=0 | 36 | 0.001 | | | *psbA-trnH＞ITS2+trnL* | |
| *psbA-trnH* | *psbA-trnH+trnL-trnF* | W^＋^=666 | W^－^=0 | 36 | 0.001 | | | *psbA-trnH＞psbA-trnH+trnL-trnF* | |
| *psbA-trnH* | *psbA-trnH+trnL* | W^＋^=665 | W^－^=1 | 36 | 0.001 | | | *psbA-trnH＞psbA-trnH+trnL* | |
| *psbA-trnH* | *trnL-trnF+trnL* | W^＋^=666 | W^－^=0 | 36 | 0.001 | | | *psbA-trnH＞trnL-trnF+trnL* | |
| *psbA-trnH* | *ITS2+psbA-trnH+trnL-trnF* | W^＋^=655 | W^－^=11 | 36 | 0.001 | | | *psbA-trnH＞ITS2+psbA-trnH+trnL-trnF* | |
| *psbA-trnH* | *ITS2+psbA-trnH+trnL* | W^＋^=663 | W^－^=3 | 36 | 0.001 | | | *psbA-trnH＞ITS2+psbA-trnH+trnL* | |
| *psbA-trnH* | *ITS2+trnL-trnF+trnL* | W^＋^=666 | W^－^=0 | 36 | 0.001 | | | *psbA-trnH＞ITS2+trnL-trnF+trnL* | |
| *psbA-trnH* | *psbA-trnH+trnL-trnF+trnL* | W^＋^=665 | W^－^=1 | 36 | 0.001 | | | *psbA-trnH＞psbA-trnH+trnL-trnF+trnL* | |
| *psbA-trnH* | *ITS2+psbA-trnH+trnL-trnF+trnL* | W^＋^=665 | W^－^=1 | 36 | 0.001 | | | *psbA-trnH＞ITS2+psbA-trnH+trnL-trnF+trnL* | |
| *trnL-trnF* | *ITS2+psbA-trnH* | W^＋^=0 | W^－^=666 | 36 | 0.001 | | | *trnL-trnF＜ITS2+psbA-trnH* | |
| *trnL-trnF* | *ITS2+trnL-trnF* | W^＋^=0 | W^－^=666 | 36 | 0.001 | | | *trnL-trnF＜ITS2+trnL-trnF* | |
| *trnL-trnF* | *ITS2+trnL* | W^＋^=0 | W^－^=666 | 36 | 0.001 | | | *trnL-trnF＜ITS2+trnL* | |
| *trnL-trnF* | *psbA-trnH+trnL-trnF* | W^＋^=0 | W^－^=666 | 36 | 0.001 | | | *trnL-trnF＜psbA-trnH+trnL-trnF* | |
| *trnL-trnF* | *psbA-trnH+trnL* | W^＋^=0 | W^－^=666 | 36 | 0.001 | | | *trnL-trnF＜psbA-trnH+trnL* | |
| *trnL-trnF* | *trnL-trnF+trnL* | W^＋^=550 | W^－^=80 | 36 | 0.001 | | | *trnL-trnF＞trnL-trnF+trnL* | |
| *trnL-trnF* | *ITS2+psbA-trnH+trnL-trnF* | W^＋^=0 | W^－^=666 | 36 | 0.001 | | | *trnL-trnF＜ITS2+psbA-trnH+trnL-trnF* | |
| *trnL-trnF* | *ITS2+psbA-trnH+trnL* | W^＋^=0 | W^－^=666 | 36 | 0.001 | | | *trnL-trnF＜ITS2+psbA-trnH+trnL* | |
| *trnL-trnF* | *ITS2+trnL-trnF+trnL* | W^＋^=0 | W^－^=666 | 36 | 0.001 | | | *trnL-trnF＜ITS2+trnL-trnF+trnL* | |
| *trnL-trnF* | *psbA-trnH+trnL-trnF+trnL* | W^＋^=0 | W^－^=666 | 36 | 0.001 | | | *trnL-trnF＜psbA-trnH+trnL-trnF+trnL* | |
| *trnL-trnF* | *ITS2+psbA-trnH+trnL-trnF+trnL* | W^＋^=0 | W^－^=666 | 36 | 0.001 | | | *trnL-trnF＜ITS2+psbA-trnH+trnL-trnF+trnL* | |
| *trnL* | *ITS2+psbA-trnH* | W^＋^=0 | W^－^=666 | 36 | 0.001 | | | *trnL＜ITS2+psbA-trnH* | |
| *trnL* | *ITS2+trnL-trnF* | W^＋^=0 | W^－^=666 | 36 | 0.001 | | | *trnL＜ITS2+trnL-trnF* | |
| *trnL* | *ITS2+trnL* | W^＋^=0 | W^－^=666 | 36 | 0.001 | | | *trnL＜ITS2+trnL* | |
| *trnL* | *psbA-trnH+trnL-trnF* | W^＋^=0 | W^－^=666 | 36 | 0.001 | | | *trnL＜psbA-trnH+trnL-trnF* | |
| *trnL* | *psbA-trnH+trnL* | W^＋^=0 | W^－^=666 | 36 | 0.001 | | | *trnL＜psbA-trnH+trnL* | |
| *trnL* | *trnL-trnF+trnL* | W^＋^=0 | W^－^=666 | 36 | 0.001 | | | *trnL＜trnL-trnF+trnL* | |
| *trnL* | *ITS2+psbA-trnH+trnL-trnF* | W^＋^=0 | W^－^=666 | 36 | 0.001 | | | *trnL＜ITS2+psbA-trnH+trnL-trnF* | |
| *trnL* | *ITS2+psbA-trnH+trnL* | W^＋^=0 | W^－^=666 | 36 | 0.001 | | | *trnL＜ITS2+psbA-trnH+trnL* | |
| *trnL* | *ITS2+trnL-trnF+trnL* | W^＋^=0 | W^－^=666 | 36 | 0.001 | | | *trnL＜ITS2+trnL-trnF+trnL* | |
| *trnL* | *psbA-trnH+trnL-trnF+trnL* | W^＋^=0 | W^－^=666 | 36 | 0.001 | | | *trnL＜psbA-trnH+trnL-trnF+trnL* | |
| *trnL* | *ITS2+psbA-trnH+trnL-trnF+trnL* | W^＋^=0 | W^－^=666 | 36 | 0.001 | | | *trnL＜ITS2+psbA-trnH+trnL-trnF+trnL* | |

**Table S2.** The average score, E-Value and Identity% in BLAST analysis

| Sequences | Average Score | E-Value | Identity% |
| --- | --- | --- | --- |
| *ITS2* | 419.5 | 9.5E-110 | 99.80 |
| *psbA-trnH* | 709.0 | 3.33E-121 | 98.67 |
| *trnL-trnF* | 603.3 | 3.75E-157 | 99.57 |
| *trnL* | 924.3 | 2.5E-173 | 98.61 |
| *ITS2+psbA-trnH* | 780.7 | 7.78E-126 | 97.51 |
| *ITS2+trnL-trnF* | 606.8 | 2.55E-161 | 99.40 |
| *ITS2+trnL* | 962.6 | 0 | 99.55 |
| *psbA-trnH+trnL-trnF* | 812.7 | 1.11E-168 | 98.93 |
| *psbA-trnH+trnL* | 962.5 | 0 | 99.54 |
| *trnL-trnF+trnL* | 963.5 | 0 | 99.67 |
| *ITS2+psbA-trnH+trnL-trnF* | 819.4 | 2.22E-168 | 98.97 |
| *ITS2+psbA-trnH+trnL* | 954.8 | 0 | 99.70 |
| *ITS2+trnL-trnF+trnL* | 961.9 | 0 | 99.60 |
| *psbA-trnH+trnL-trnF+trnL* | 964.0 | 0 | 99.69 |
| *ITS2+psbA-trnH+trnL-trnF+trnL* | 961.9 | 0 | 99.60 |

**Table S3.** The GenBank accession accession numbers for sequences

| Species Name | Sample No | ITS2 | psbA-trnH | trnL-trnF | trnL |
| --- | --- | --- | --- | --- | --- |
| *Syringa oblata* | 1 | OQ702506 | PQ642050 | PQ642077 | PQ642104 |
| *Syringa oblata* | 2 | OQ804782 | PQ642051 | PQ642078 | PQ642105 |
| *Syringa oblata* | 3 | OQ804789 | PQ642052 | PQ642079 | PQ642106 |
| *Syringa vulgaris* | 4 | OQ708343 | PQ642053 | PQ642080 | PQ642107 |
| *Syringa vulgaris* | 5 | OQ708347 | PQ642054 | PQ642081 | PQ642108 |
| *Syringa vulgaris* | 6 | OQ702503 | PQ642055 | PQ642082 | PQ642109 |
| *Syringa wolfii* | 7 | OQ702507 | PQ642056 | PQ642083 | PQ642110 |
| *Syringa wolfii* | 8 | OQ804786 | PQ642057 | PQ642084 | PQ642111 |
| *Syringa wolfii* | 9 | OQ804784 | PQ642058 | PQ642085 | PQ642112 |
| *Syringa villosa* | 10 | OQ702502 | PQ642059 | PQ642086 | PQ642113 |
| *Syringa villosa* | 11 | OQ804787 | PQ642060 | PQ642087 | PQ642114 |
| *Syringa villosa* | 12 | OQ804790 | PQ642061 | PQ642088 | PQ642115 |
| *Syringa josikaea* | 13 | OQ702500 | PQ642062 | PQ642089 | PQ642116 |
| *Syringa josikaea* | 14 | OQ804816 | PQ642063 | PQ642090 | PQ642117 |
| *Syringa josikaea* | 15 | OQ804809 | PQ642064 | PQ642091 | PQ642118 |
| *Syringa reticulata subsp. pekinensis* | 16 | PQ722901 | PQ642065 | PQ642092 | PQ642119 |
| *Syringa reticulata subsp. pekinensis* | 17 | PQ722902 | PQ642066 | PQ642093 | PQ642120 |
| *Syringa reticulata subsp. pekinensis* | 18 | PQ722903 | PQ642067 | PQ642094 | PQ642121 |
| *Syringa reticulata subsp. amurensis* | 19 | OQ832596 | PQ642068 | PQ642095 | PQ642122 |
| *Syringa reticulata subsp. amurensis* | 20 | OQ804781 | PQ642069 | PQ642096 | PQ642123 |
| *Syringa reticulata subsp. amurensis* | 21 | OQ804780 | PQ642070 | PQ642097 | PQ642124 |
| *Syringa pubescens subsp. patula Palibin* | 22 | PQ621266 | PQ642071 | PQ642098 | PQ642125 |
| *Syringa pubescens subsp. patula Palibin* | 23 | PQ621269 | PQ642072 | PQ642099 | PQ642126 |
| *Syringa pubescens subsp. patula Palibin* | 24 | PQ621270 | PQ642073 | PQ642100 | PQ642127 |
| *Syringa meyeri* | 25 | PQ621265 | PQ642074 | PQ642101 | PQ642128 |
| *Syringa meyeri* | 26 | PQ621267 | PQ642075 | PQ642102 | PQ642129 |
| *Syringa meyeri* | 27 | PQ621268 | PQ642076 | PQ642103 | PQ642130 |

**Table S4.** Sequences and primers information

| **No.** | **Sequences** | **Primers（3'-5'）** | **References** |
| --- | --- | --- | --- |
| 1 | *ITS2* | F: YGACTCTCGGCAACGGATA | [1] |
|  |  | R: RGTTTCTTTTCCTCCGCTTA |  |
| 2 | *psbA-trnH* | F: GTTATGCATGAACGTAATGCTC | [2] |
|  |  | R: CGCGCATGGTGGATTCACAAATC |  |
| 3 | *trnL-trnF* | F: GGTTCAAGTCCCTCTATCCC | [3] |
|  |  | R: ATTTGAACTGGTGACACGAG |  |
| 4 | *trnL* | F: CGAAATCGGTAGACGCTACG- | [4] |
|  |  | R: GGGGATAGAGGGACTTGAAC |  |

1. Hu, S.J.; Hu, H.Y.; Gao, H.; Liu, X.; Chen, S.L. DNA barcoding and rapid identification of the precious herb Herba Anoectochili. *Chin. J. Nat. Med.* **2019**, *17*, 738-745.

2. Srirama, R.; Senthilkumar, U.; Sreejayan, N.; Ravikanth, G.; Gurumurthy, B.R.; Shivanna, M.B.; Sanjappa, M.; Ganeshaiah, K.N.; Shaanker, R.U. Assessing species admixtures in raw drug trade of Phyllanthus, a hepato-protective plant using molecular tools. *J. Ethnopharmacol.* **2010**, *130*, 208-215.

3. Taberlet, P.; Gielly, L.; Pautou, G.; Bouvet, J. Universal primers for amplification of three non-coding regions of chloroplast DNA. *Plant molecular biology* **1991**, *17*, 1105-1109.

4. Gielly, L.; Yuan, Y.M.; Kupfer, P.; Taberlet, P. Phylogenetic use of noncoding regions in the genus Gentiana L.: chloroplast trnL (UAA) intron versus nuclear ribosomal internal transcribed spacer sequences. *Molecular phylogenetics and evolution* **1996**, *5*, 460-466.

**8 S.wolfii**

**9 S.wolfii**

**7 S.wolfii**

**11 S.villosa**

**10 S.villosa**

**12 S.villosa**

**13 S.josikaea**

**14 S.josikaea**

**15 S.josikaea**

**17 S.reticulata subsp. pekinensis**

**18 S.reticulata subsp. pekinensis**

**16 S.reticulata subsp. pekinensis**

**21 S.reticulata subsp. amurensis**

**19 S.reticulata subsp. amurensis**

**20 S.reticulata subsp. amurensis**

**23 S.pubescens subsp. patula Palibin**

**24 S.pubescens subsp. patula Palibin**

**22 S.pubescens subsp. patula Palibin**

**25 S.meyeri**

**26 S.meyeri**

**27 S.meyeri**

**1 S.oblata**

**2 S.oblata**

**3 S.oblata**

**4 S.vulgaris**

**5 S.vulgaris**

**6 S.vulgaris**

**35**

**100**

**17**

**18**

**94**

**31**

**96**

**69**

**31**

**100**

**68**

**100**

**54**

**88**

**31**

**100**

**34**

**99**

**100**

**81**

**100**

**32**

**100**

**88**

**Supplementary Figure S1.** NJ tree constructed based on *ITS2+psbA-trnH*

**23 S.pubescens subsp. patula Palibin**

**24 S.pubescens subsp. patula Palibin**

**22 S.pubescens subsp. patula Palibin**

**25 S.meyeri**

**26 S.meyeri**

**27 S.meyeri**

**17 S.reticulata subsp. pekinensis**

**18 S.reticulata subsp. pekinensis**

**16 S.reticulata subsp. pekinensis**

**21 S.reticulata subsp. amurensis**

**19 S.reticulata subsp. amurensis**

**20 S.reticulata subsp. amurensis**

**14 S.josikaea**

**15 S.josikaea**

**13 S.josikaea**

**12 S.villosa**

**11 S.villosa**

**10 S.villosa**

**7 S.wolfii**

**8 S.wolfii**

**9 S.wolfii**

**4 S.vulgaris**

**5 S.vulgaris**

**6 S.vulgaris**

**3 S.oblata**

**1 S.oblata**

**2 S.oblata**

**32**

**89**

**31**

**89**

**97**

**35**

**99**

**69**

**99**

**83**

**60**

**31**

**98**

**31**

**87**

**27**

**26**

**93**

**75**

**71**

**67**

**25**

**24**

**100**

**Supplementary Figure S2.** NJ tree constructed based on *ITS2+trnL-trnF*

**27 S.meyeri**

**25 S.meyeri**

**26 S.meyeri**

**22 S.pubescens subsp. patula Palibin**

**23 S.pubescens subsp. patula Palibin**

**24 S.pubescens subsp. patula Palibin**

**17 S.reticulata subsp. pekinensis**

**18 S.reticulata subsp. pekinensis**

**16 S.reticulata subsp. pekinensis**

**21 S.reticulata subsp. amurensis**

**19 S.reticulata subsp. amurensis**

**20 S.reticulata subsp. amurensis**

**14 S.josikaea**

**15 S.josikaea**

**13 S.josikaea**

**8 S.wolfii**

**9 S.wolfii**

**7 S.wolfii**

**10 S.villosa**

**11 S.villosa**

**12 S.villosa**

**4 S.vulgaris**

**5 S.vulgaris**

**6 S.vulgaris**

**3 S.oblata**

**1 S.oblata**

**2 S.oblata**

**32**

**96**

**27**

**88**

**33**

**99**

**70**

**95**

**91**

**58**

**33**

**93**

**27**

**86**

**27**

**79**

**59**

**69**

**67**

**66**

**20**

**22**

**100**

**Supplementary Figure S3.** NJ tree constructed based on *ITS2+trnL*

**8 S.wolfii**

**9 S.wolfii**

**7 S.wolfii**

**10 S.villosa**

**11 S.villosa**

**12 S.villosa**

**16 S.reticulata subsp. pekinensis**

**17 S.reticulata subsp. pekinensis**

**18 S.reticulata subsp. pekinensis**

**13 S.josikaea**

**14 S.josikaea**

**15 S.josikaea**

**19 S.reticulata subsp. amurensis**

**20 S.reticulata subsp. amurensis**

**21 S.reticulata subsp. amurensis**

**2 S.oblata**

**3 S.oblata**

**1 S.oblata**

**5 S.vulgaris**

**6 S.vulgaris**

**4 S.vulgaris**

**23 S.pubescens subsp. patula Palibin**

**24 S.pubescens subsp. patula Palibin**

**22 S.pubescens subsp. patula Palibin**

**25 S.meyeri**

**26 S.meyeri**

**27 S.meyeri**

**34**

**100**

**32**

**99**

**29**

**98**

**100**

**32**

**100**

**51**

**34**

**100**

**31**

**100**

**93**

**31**

**97**

**60**

**58**

**33**

**99**

**72**

**30**

**91**

**Supplementary Figure S4.** NJ tree constructed based on *psbA-trnH+trnL-trnF*

**8 S.wolfii**

**9 S.wolfii**

**7 S.wolfii**

**16 S. reticulata subsp. pekinensis**

**17 S. reticulata subsp. pekinensis**

**18 S. reticulata subsp. pekinensis**

**10 S.villosa**

**11 S.villosa**

**12 S.villosa**

**13 S.josikaea**

**14 S.josikaea**

**15 S.josikaea**

**19 S.reticulata subsp. amurensis**

**20 S.reticulata subsp. amurensis**

**21 S.reticulata subsp. amurensis**

**2 S.oblata**

**3 S.oblata**

**1 S.oblata**

**5 S.vulgaris**

**6 S.vulgaris**

**4 S.vulgaris**

**22 S.pubescens subsp. patula Palibin**

**23 S.pubescens subsp. patula Palibin**

**24 S.pubescens subsp. patula Palibin**

**25 S.meyeri**

**26 S.meyeri**

**27 S.meyeri**

**33**

**100**

**33**

**100**

**34**

**100**

**36**

**100**

**32**

**96**

**97**

**68**

**34**

**100**

**92**

**32**

**99**

**36**

**35**

**100**

**58**

**55**

**33**

**97**

**Supplementary Figure S5.** NJ tree constructed based on *psbA-trnH+trnL*

**11 S.villosa**

**12 S.villosa**

**10 S.villosa**

**13 S.josikaea**

**14 S.josikaea**

**15 S.josikaea**

**7 S.wolfii**

**8 S.wolfii**

**9 S.wolfii**

**24 S.pubescens subsp. patula Palibin**

**22 S.pubescens subsp. patula Palibin**

**23 S.pubescens subsp. patula Palibin**

**25 S.meyeri**

**26 S.meyeri**

**27 S.meyeri**

**4 S.vulgaris**

**5 S.vulgaris**

**6 S.vulgaris**

**1 S.oblata**

**2 S.oblata**

**3 S.oblata**

**19 S.reticulata subsp. amurensis**

**20 S.reticulata subsp. amurensis**

**21 S.reticulata subsp. amurensis**

**16 S.reticulata subsp. pekinensis**

**17 S.reticulata subsp. pekinensis**

**18 S.reticulata subsp. pekinensis**

**33**

**99**

**33**

**95**

**32**

**94**

**23**

**66**

**21**

**20**

**98**

**27**

**82**

**71**

**27**

**25**

**80**

**49**

**22**

**62**

**64**

**41**

**17**

**49**

**Supplementary Figure S6.** NJ tree constructed based on *trnL-trnF+trnL*

**17 S.reticulata subsp. pekinensis**

**18 S.reticulata subsp. pekinensis**

**16 S.reticulata subsp. pekinensis**

**21 S.reticulata subsp. amurensis**

**19 S.reticulata subsp. amurensis**

**20 S.reticulata subsp. amurensis**

**23 S.pubescens subsp. patula Palibin**

**24 S.pubescens subsp. patula Palibin**

**22 S.pubescens subsp. patula Palibin**

**25 S.meyeri**

**26 S.meyeri**

**27 S.meyeri**

**14 S.josikaea**

**15 S.josikaea**

**13 S.josikaea**

**8 S.wolfii**

**9 S.wolfii**

**7 S.wolfii**

**10 S.villosa**

**11 S.villosa**

**12 S.villosa**

**4 S.vulgaris**

**5 S.vulgaris**

**6 S.vulgaris**

**3 S.oblata**

**2 S.oblata**

**1 S.oblata**

**32**

**100**

**67**

**66**

**22**

**24**

**65**

**93**

**96**

**32**

**94**

**29**

**89**

**96**

**59**

**100**

**32**

**97**

**28**

**85**

**88**

**93**

**15**

**51**

**Supplementary Figure S7.** NJ tree constructed based on *ITS2+psbA-trnH+trnL-trnF*

**8 S.wolfii**

**9 S.wolfii**

**7 S.wolfii**

**16 S.reticulata subsp. pekinensis**

**17 S.reticulata subsp. pekinensis**

**18 S.reticulata subsp. pekinensis**

**10 S.villosa**

**11 S.villosa**

**12 S.villosa**

**13 S.josikaea**

**14 S.josikaea**

**15 S.josikaea**

**19 S.reticulata subsp. amurensis**

**20 S.reticulata subsp. amurensis**

**21 S.reticulata subsp. amurensis**

**26 S.meyeri**

**27 S.meyeri**

**25 S.meyeri**

**22 S.pubescens subsp. patula Palibin**

**23 S.pubescens subsp. patula Palibin**

**24 S.pubescens subsp. patula Palibin**

**2 S.oblata**

**3 S.oblata**

**1 S.oblata**

**4 S.vulgaris**

**5 S.vulgaris**

**6 S.vulgaris**

**31**

**100**

**34**

**100**

**33**

**100**

**33**

**100**

**34**

**98**

**99**

**52**

**31**

**100**

**94**

**34**

**99**

**34**

**25**

**100**

**40**

**32**

**32**

**Supplementary Figure S8.** NJ tree constructed based on *psbA-trnH+trnL-trnF+trnL*
