# Supplementary figures and images for: Morphological description and DNA barcoding research of nine Syringa species
Source: Front Genet. 2025 Feb 26;16:1544062. doi: 10.3389/fgene.2025.1544062 (PMC11897579; doi:10.3389/fgene.2025.1544062)

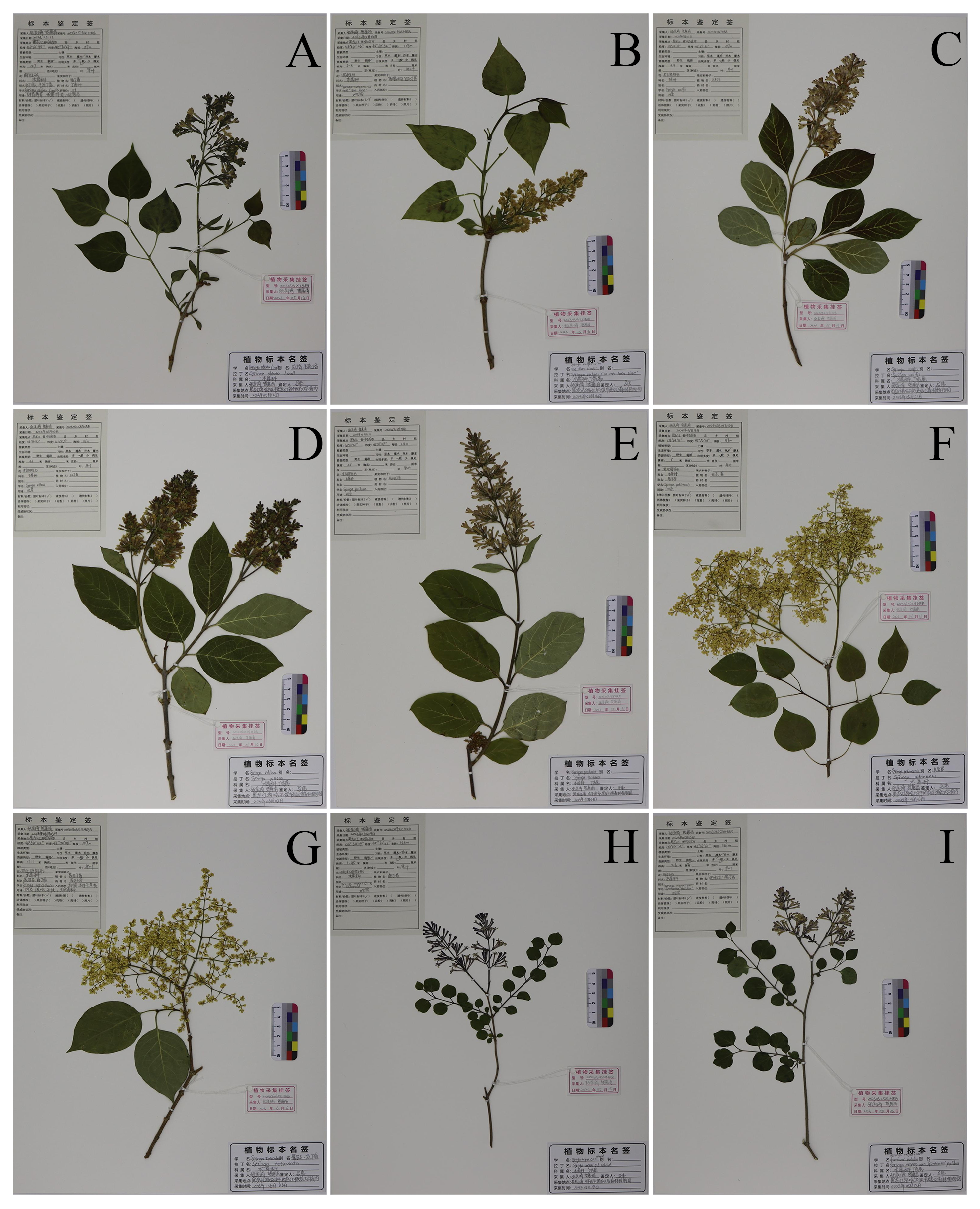

Supplement: Supplementary file 2 [file Image1.tif]
